# Eukaryote specific RNA and protein features facilitate assembly and catalysis of H/ACA snoRNPs

**DOI:** 10.1093/nar/gkab177

**Published:** 2021-04-06

**Authors:** Sven Trucks, Gerd Hanspach, Martin Hengesbach

**Affiliations:** Institute for Organic Chemistry and Chemical Biology, Goethe-University Frankfurt, Max-von-Laue-Str. 7, 60438 Frankfurt, Germany; Institute for Organic Chemistry and Chemical Biology, Goethe-University Frankfurt, Max-von-Laue-Str. 7, 60438 Frankfurt, Germany; Institute for Organic Chemistry and Chemical Biology, Goethe-University Frankfurt, Max-von-Laue-Str. 7, 60438 Frankfurt, Germany

## Abstract

H/ACA Box ribonucleoprotein complexes (RNPs) play a major role in modification of rRNA and snRNA, catalyzing the sequence specific pseudouridylation in eukaryotes and archaea. This enzymatic reaction takes place on a substrate RNA recruited via base pairing to an internal loop of the snoRNA. Eukaryotic snoRNPs contain the four proteins Nop10, Cbf5, Gar1 and Nhp2, with Cbf5 as the catalytic subunit. In contrast to archaeal H/ACA RNPs, eukaryotic snoRNPs contain several conserved features in both the snoRNA as well as the protein components. Here, we reconstituted the eukaryotic H/ACA RNP containing snR81 as a guide RNA *in vitro* and report on the effects of these eukaryote specific features on complex assembly and enzymatic activity. We compare their contribution to pseudouridylation activity for stand-alone hairpins versus the bipartite RNP. Using single molecule FRET spectroscopy, we investigated the role of the different eukaryote-specific proteins and domains on RNA folding and complex assembly, and assessed binding of substrate RNA to the RNP. Interestingly, we found diverging effects for the two hairpins of snR81, suggesting hairpin-specific requirements for folding and RNP formation. Our results for the first time allow assessing interactions between the individual hairpin RNPs in the context of the full, bipartite snoRNP.

## INTRODUCTION

In eukaryotes, small nucleolar RNAs (snoRNAs) are involved in modification and processing of rRNA and snRNA (1–3). SnoRNAs can be separated into two categories; box C/D RNAs mediating 2′-*O*-methylation, and box H/ACA RNAs involved in the isomerization of uridine to pseudouridine (Ψ). Both modifications are highly conserved and abundant in eukaryotes as well as archaea (3,4).

To confer enzymatic activity, all snoRNAs require additional proteins to form a ribonucleoprotein (RNP) complex, wherein the RNA assumes a guiding function by binding the target RNA via base pairing, ensuring site-specific modification (5).

**Figure 1.**
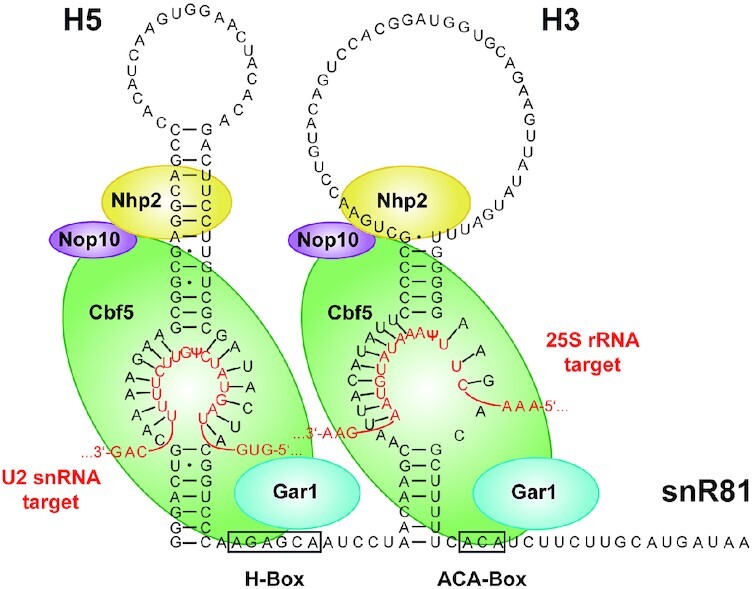
RNP complex formed with snoRNA snR81 (black sequence). The bipartite architecture is governed by the two RNA hairpins H5 and H3, which each bind one set of the core proteins Nhp2 (yellow), Nop10 (violet), the pseudouridylase Cbf5 (green), and Gar1 (blue). The H/ACA denomination arises from the two sequence motifs on the 3′ end of each hairpin, termed H-Box and ACA-Box. Each hairpin binds its substrate RNA (red sequence) by complementary base-pairing, with H5 targeting U42 of the U2 snRNA, and H3 targeting U1051 of the 25S rRNA.

Ψ—the most abundant modification found in all cellular RNAs—can be introduced in an RNA independent fashion by stand-alone proteins, or in an RNA dependent manner by H/ACA RNPs (6–8). For RNA-guided pseudouridylation, box H/ACA RNAs form hairpin-bulge-hairpin motifs (one to three in archaea, two in eukaryotes), each binding a set of four proteins: Nhp2 (L7Ae in Archaea), Nop10, Gar1, and Cbf5 (Dyskerin or NAP57 in mammals) (Figure 1). Although sequences of H/ACA snoRNAs differ, in most cases both hairpins contain a pseudouridylation pocket, where the target RNA is recruited, isomerized and released, as well as the genetically conserved H-box (ANANNA) or ACA trinucleotide box. Eukaryotic box H/ACA RNPs are commonly found in nucleoli and Cajal bodies, where Ψ is generated. Apart from pseudouridylation, other functions of these RNA-protein complexes have been identified, such as pre-rRNA processing (6) or stabilization of telomerase RNA (9), fulfilling essential roles for ribosome biogenesis and telomere maintenance. Unsurprisingly, malfunction of H/ACA RNPs has been linked to several diseases like Dyskeratosis congenita—a bone marrow failure syndrome—and several types of cancer (10–12), showing the importance to fully understand this enzyme class.

To date most structural data on these RNPs stems from crystallization studies of archaeal single hairpin H/ACA RNPs. Full and partial crystal structures of H/ACA RNPs from thermophilic organisms have given plentiful insights into structure and function of RNA guided pseudouridylation (13–16). These structures all show a conserved RNP architecture: L7Ae, Nop10 and Cbf5 (the reactive enzyme) directly interact with the guide RNA, while Gar1 is bound to Cbf5 only. The PUA domain of Cbf5 anchors the ACA-box of the guide RNA, while the thumb loop interacts with the substrate to form a reactive state. Fluorescence correlation spectroscopy experiments showed that Gar1 destabilizes the product complex and facilitates product release (17).

This detailed structural knowledge is derived from archaeal RNA guided Ψ-synthases, while eukaryotic pseudouridylation remains less well characterized, as only limited structural data is available for eukaryotic H/ACA RNPs. So far, only a crystal structure of a ternary Nop10, Cbf5 and Gar1 (NCG) subcomplex—comprising only the core domains of Cbf5 and Gar1 (18)—and an NMR structure of Nhp2 (19) from yeast (*Saccharomyces cerevisiae*) have been solved, showing high homology between the archaeal and eukaryotic core domains of Cbf5 and Gar1, as well as Nhp2 and L7Ae. A recent cryo-EM structure containing the H/ACA RNP of human telomerase has been solved (20), but since the RNA component hTR fulfils distinctly different cellular roles and has not been established as a pseudouridylation guide RNA, the structural conclusions in regard to eukaryotic pseudouridylation may be of limited value.

Comparing eukaryotic and archaeal H/ACA RNPs, Nhp2 and L7Ae interact in different ways with the H/ACA RNA. As a kink-turn binding protein, L7Ae strongly interacts with the conserved K-loop in archaeal H/ACA RNAs even in absence of Nop10, Cbf5 and Gar1 (21), but shows limited binding affinity to the other proteins when the guide RNA is missing (22). In contrast, Nhp2 shows non-specific binding to RNA stem-loops in the absence of the other proteins, while specific binding to the snoRNA seems to be governed by formation of an Nhp2-Nop10-Cbf5 subcomplex before the RNP is assembled (23). Along the same line, Nhp2 exhibits a 1000-fold higher binding affinity to the snR34 guide RNA of yeast when the other proteins are present (24).

Gar1, which is the only protein to not directly interact with the guide RNA, has been shown to act as a mediator between the open and closed state of the archaeal Cbf5 thumb-loop, controlling product release and therefore multiple turnover enzymatic activity in archaea (14,16,17,25). In contrast to its archaeal counterpart, eukaryotic Gar1 contains several glycine-arginine rich (RGG) domains both at its N- and C-terminus. While the role of these RGG domains is elusive in H/ACA RNPs, in other RGG domain containing proteins, they have been observed to enhance the function of primary RNA binding motives and therefore play an accessory role in RNA binding (26–31). They furthermore are involved in liquid-liquid phase separation processes (32).

So far, reconstitution of active H/ACA RNPs from recombinant proteins has been reported for snR5 and more recently for snR34 guide RNAs (18,24). Both RNAs conferred high catalytic turnover rates *in vitro* for the fully assembled RNPs in excess of 5′- or 3′-substrates. In both cases, Ψ formation was consistently faster for the 3′-hairpin. For snR5, each standalone hairpin facilitates pseudouridylation, even though isomerization rates are reduced in absence of the other hairpin, indicating that there are molecular interactions between the two hairpins that promote catalytic activity. For both snR5 and snR34, omission of Nhp2 leads to losses in substrate turnover rates. However, the impact on isomerization activity varies for each snoRNA and individual hairpin. While for the snR34 RNP removal of Nhp2 significantly reduces pseudouridylation for both hairpins, for the snR5 complex only the 5′-hairpins seems to be severely affected by absence of the RNA binding protein.

Despite these biochemical analyses, the exact function of eukaryotic specific features in H/ACA RNA guided pseudouridylation remains elusive. This holds true for both eukaryote specific protein domains and the bipartite RNA architecture. We therefore assessed the contribution of each of these features alone, and in combination, to identify interactions between them. This study therefore for the first time provides a detailed analysis of both RNA folding and interactions within a full bipartite snoRNP.

## MATERIALS AND METHODS

### Generation of protein constructs

#### Expression and purification of Cbf5, Nop10 and Gar1

Genes of Cbf5 (restriction sites: NcoI/EcoRI), Nop10 (restriction sites: NdeI/XhoI) and Gar1 (restriction sites: NdeI/EcoRI) from *S. cerevisiae* were ordered codon optimized from Eurofins Genomics. Cbf5 and Nop10 were cloned into MCS1 and 2 of a pET-Duet vector, respectively. Gar1 was cloned into a pET-28b vector.

All three proteins were co-expressed from *Escherichia coli* BL21 (DE3) cells. The cells containing both plasmids were inoculated using 1 l Terrific Broth medium, containing ampicillin (100 μg ml^−1^) and kanamycin (30 μg ml^−1^) at an OD_600_ ∼0.1. Cells were incubated (37°C, 130 rpm) to an OD_600_ ∼0.8 and cooled on ice for 15 min. Expression was induced by adding 0.1 mM isopropyl β-d-1-thiogalactopyranoside (IPTG) and cells were incubated for ∼20 h (20°C, 130 rpm) before being harvested (8000 × g, 30 min, 4°C) and resuspended in 30 ml buffer A [50 mM Tris/HCl (pH 8.0), 0.5 M NaCl, 10% glycerol (v/v), 15 mM imidazole, 7 mM 2-mercapthoethanol], supplemented with EDTA-free protease inhibitor cocktail (Roche), 200 μg of RNase A (Invitrogen) and 20 U of Turbo DNase (Thermo Fisher Scientific). Lysis was done via sonication and lysate separated from cell debris by centrifugation (21 000 × g, 1 h, 4°C). Lysate was supplemented with polyethylenimine (PEI) [0.017% (w/v)], incubated at 4°C for 15 min and precipitated nucleic acids removed via centrifugation (10 000 × g, 20 min, 4°C). PEI treatment was repeated once, and the supernatant was filtered (0.2 μm) and loaded onto a Ni^2+^-NTA column (HisTrap HP, GE Healthcare) preequilibrated with buffer A. The loaded column was washed with buffer A and protein elution was done with a linear gradient to buffer B [50 mM Tris/HCl (pH 8.0), 0.5 M NaCl, 10% glycerol (v/v), 500 mM imidazole, 7 mM 2-mercapthoethanol] with an Äkta start system. Protein containing fractions (absorption at 280 nm) were analyzed by 15% (w/v) SDS-PAGE, pooled and concentrated to a volume of ∼100 μl (VivaSpin 20, 50 kDa molecular weight cutoff, PES (Sartorius)). Further purification was performed via SEC (Superdex 200 10/300 GL column (GE Healthcare), with buffer C [50 mM Tris/HCl (pH 8.0), 0.5 M NaCl, 10% glycerol (v/v)] on an Äkta purifier 900 system. Protein containing fractions (absorption at 280 nm) again were analyzed by 15% (w/v) SDS-PAGE (Supplementary Figure S1), pooled and concentrated to a volume of ∼100 μl. The protein concentration was determined with a Nanodrop one UV−Vis spectrophotometer (Thermo Fisher Scientific, extinction coefficient ϵ = 62 800 M^−1^ cm^−1^ at 280 nm).

#### Expression and purification of Nhp2

The gene coding for Nhp2 from *Saccharomyces cerevisiae* was ordered codon optimized from EurofinsGenomics and cloned (restriction sites: NdeI/XhoI) into a pET-24b vector. Mutation S82W was introduced as described below and protein was expressed from *Escherichia coli* BL21 (DE3) cells.

The cells were inoculated with 1 l Terrific Broth medium, containing kanamycin (30 μg ml^−1^) at an OD_600_ ∼0.1. Protein expression and purification was done accordingly to the protocol described for NCG proteins. Additionally, after the first Ni^2+^-NTA, protein containing fractions (absorption at 280 nm) were analyzed by 15% (w/v) SDS-PAGE, pooled and incubated with 400 μg RNase A (20°C, 16 h) to remove copurified contaminating RNA. Afterwards, the protein solution was diluted to an imidazole concentration of ∼20 mM and RNase A removed via a second Ni^2+^-NTA column purification. For concentration of the protein samples, VivaSpin 20 with a 10 kDa molecular weight cutoff, PES (Sartorius), for size exclusion Superdex 75 10/300 GL column (GE Healthcare) and for concentration determination ϵ = 9970 M^−1^ cm^−1^ were used (Supplementary Figure S1).

#### Introduction of codon at desired position

The introduction of a tryptophan (TGG) and an amber stop codon (TAG) into Nhp2 at positions S82 (19), K48 and K37, respectively was performed via site-directed mutagenesis employing the QuikChange Site-directed Mutagenesis Kit (Agilent Genomics). DNA oligonucleotides acting as mutagenesis primers (Supplementary Table S1) were designed according to manufacturer protocols and purchased from Eurofins Genomics (Munich, Germany).

#### Synthesis of propargyl-lysine

Unnatural amino acid propargyl-lysine (PrK) was synthesized according to literature (33) and analyzed via NMR.

#### Expression and purification of Nhp2K48PrK and Nhp2K37PrK mutants

Protein Nhp2 (on pET-24b plasmid) from *S. cerevisiae* and the pEVOL plasmid (encoding the WT pyrrolysyl tRNA/pyrrolysyl-tRNA synthetase pair from *Methanosarcina mazei* (33)) were co-expressed from *E. coli* BL21 (DE3) cells.

The cells containing both plasmids were inoculated with 1 l Terrific Broth medium, containing kanamycin (30 μg ml^−1^) and chloramphenicol (34 μg ml^−1^) at an OD_600_ ∼0.1. Cells were incubated (37°C, 130 rpm) for 1 h, supplemented with 2.5 mM PrK and incubated for an additional 1 h. Afterwards, arabinose was added to a final concentration of 0.2% (w/v) to induce the pEVOL system. Cells were incubated for an additional 5 h before being put on ice for 15 min. Nhp2 expression was induced by adding 0.1 mM IPTG and cells were incubated for ∼48 h (20°C, 130 rpm) before being harvested (8000 × g, 30 min, 4°C). Protein purification was done according to the protocol for Nhp2 (Supplementary Figure S1). PrK incorporation was verified via MALDI mass spectrometry (Supplementary Figure S2).

#### Mass spectrometry

Protein was desalted with a C18 resin pipet tip (ZipTip, Millipore) prior to measurement. MALDI-TOF was performed by the mass spectrometry service at the Goethe-University Frankfurt.

### Pseuoduridylation activity assays

#### Preparation and purification of snoRNAs

Synthesis of the DNA transcription templates (Supplementary Table S1) was performed by PCR. SnoRNAs were prepared by transcription from DNA templates (Supplementary Table S1), using 3.75 mM of each NTP (ATP, CTP, GTP, UTP), 15 mM GMP, 30 mM magnesium acetate, 100 mM Tris/HCl, 10 mM DTT, 3 mM spermidine, 0.01 μg/μl of DNA template and 24 ng/μl of homemade T7 RNA polymerase. Samples were incubated at 37°C for 5 h. Afterwards, pyrophosphate precipitation was dissolved with 40 mM EDTA, DNA was digested with 0.02 U/μl of Turbo DNase (Thermo Fisher Scientific) at 37°C for 1 h and enzymes were removed via phenol extraction (1 volume aqua phenol, 3 volumes diethylether). RNA was precipitated with 0.5 M ammonium acetate and 2.5 volumes ethanol, resuspended in H_2_O/formamide (1:1, v/v) and the product was separated from side products by 12% (w/v) denaturing urea−PAGE. Product was identified via UV shadowing, excised and eluted from the gel in 0.5 M ammonium acetate by shaking at 16°C overnight. RNA was precipitated with ethanol (2.5 volumes), resuspended in H_2_O and the concentration was determined with a Nanodrop one UV−Vis spectrophotometer (Thermo Fisher Scientific).

#### Generation of ^32^P-labeled substrate RNAs via splinted ligation

Oligonucleotides for generation of ^32^P-labeled substrate RNAs were purchased from EurofinsGenomics (Supplementary Table S2). For generation of H5 substrate H5-S, RNA oligonucleotide S5-1 was resuspended in H_2_O and ^32^P-labeling was achieved via phosphorylation in the presence of γ-^32^P-ATP (Hartmann Analytic) in 1xT4 buffer with T4-PNK (20 U) at 37°C for 30 min. Afterwards, non-radioactive ATP was added (10 mM) and phosphorylation was continued for 30 min. Excess ATP was removed via Microspin G-25 column (GE Healthcare). Labeled oligonucleotide S5-1, oligonucleotide S5-2 and DNA splint S5-S were added to equimolar amounts (50 μM) and hybridized in 1× T4 ligase buffer by heating to 75°C for 2 min and slow cooling to room temperature. Splint ligation was performed with T4 DNA Ligase in 1× T4 ligase buffer at 16°C overnight. H5-S was digested with addition of 9 U Turbo DNase (Thermo Fisher Scientific) at 37°C for 1.5 h. The ligation product was separated from unreacted oligonucleotides by 15% (w/v) denaturing urea−PAGE. The ligation product was identified via a storage phosphor screen (GE Healthcare), excised and eluted from the gel in 0.5 M ammonium acetate by shaking at 16°C overnight. RNA was precipitated with ethanol (2.5 volumes), and resuspended in H_2_O. RNA concentration was calculated from ^32^P radioactivity, and verified after decay with a Nanodrop one UV−Vis spectrophotometer (Thermo Fisher Scientific). For generation of H3 substrate H3-S, the same protocol was used with oligonucleotides S3-1, S3-2 and splint S3-S.

#### Assay conditions and quantification of generated pseudouridine

Reconstitution of RNP complexes was done in psi buffer [12.5 mM Tris/HCl (pH 8.0), 250 mM NaCl] with 100 nM snoRNA and 1 μM of proteins for the H5 and H3 constructs and 2 μM proteins for the full-length (FL) construct for multiple turnover conditions, as well as 1 μM snoRNA and 2 μM of proteins for the H5 and H3 constructs and 4 μM proteins for the FL construct for single turnover conditions. 100 nM of ^32^P-labeled substrate RNA and 3.9 μM of unlabeled substrate RNA (ordered from Biomers or Dharmacon) for multiple turnover conditions and 500 nM of ^32^P-labeled substrate RNA for single turnover conditions were added to the reconstituted RNPs, and these were immediately incubated at 30°C. At different time points, samples of 20 μl were taken from the reaction tubes and added to water saturated phenol to stop enzyme activity. The aqueous phase was washed with water saturated diethyl ether and RNA was precipitated with 0.5 M sodium acetate and ethanol. RNA was resuspended in buffer P1 (20 mM ammonium acetate, 500 μM zinc chloride) and 0.25 U P1 endonuclease (Sigma Aldrich) was added. Samples were incubated at 55°C for 4 h to fully digest any RNA to mononucleotides. Afterwards, 2–3 μl of each sample were spotted on a thin layer chromatography cellulose plate (Merck) and thin layer chromatography was performed with buffer TLC [70% 2-propanol, 15% H_2_O, 15% conc. HCl (v/v)]. TLC plates were exposed on a storage phosphor screen (GE Healthcare) for 24–48 h and scanned at a Typhoon 9400 (GE Healthcare) (Supplementary Figure S3). Spots were assigned according to known *R*_f_ values (34). Image analysis and integration of peaks was performed with ImageJ (version 1.50i), data points were fitted to equation $\Psi \; = \frac{{{\Psi _{max}} \cdot t}}{{{t_{{\Psi _{1/2}}}} + \;t}}\;$. Starting turnover rates *v*_start_ were determined by linear regression over the first 5 min of fitted data.

### Generation of fluorophore-labeled constructs

#### Protein Labeling via CuAAC and purification

Dye labeling of Nhp2 was achieved with purified, site-specifically PrK-modified protein (100 μM) and Sulfo-Cy3-azide (250 μM, Jena Bioscience) in buffer C, containing 500 μM CuSO_4_, 2.5 mM tris(3-hydroxypropyltriazolylmethyl)amine (THPTA, Sigma Aldrich), 5 mM aminoguanidine, and 5 mM sodium ascorbate (freshly prepared) at 37°C for 4 h. SEC [Superdex 75 10/300 GL column (GE Healthcare), flowrate 0.5 ml min^−1^] with buffer C was performed to remove unreacted dye, labeled protein containing fractions (absorption at 280 and 550 nm) were analyzed by 15% (w/v) SDS-PAGE, pooled and concentrated to a volume of ∼100 μl (Supplementary Figure S1). A labeling efficiency of 40–50% was determined with a Nanodrop one UV−vis spectrophotometer (Thermo Fisher Scientific).

#### Generation of fluorophore-labeled RNA via splinted ligation

smFRET suitable RNAs were obtained by individual labeling of purchased amino-modified oligomers (Dharmacon) (Supplementary Table S3), DNA-splinted ligation of the labeled oligos and subsequent PAGE purification. For synthesis of acceptor and donor/acceptor fluorophore labeled RNA constructs, each RNA was divided into three oligomers.

Labeling of RNA oligomers was performed using the amine-reactive dyes Cy3 or Cy5 respectively (Amersham CyDye Mono-Reactive Dye Packs, GE Healthcare). 30 nmol of the oligomer containing a modification site were ethanol precipitated and the dried pellet was dissolved in 20 μl of a freshly prepared 0.1 M NaHCO_3_ solution. The dye was dissolved in 20 μl DMSO and added to RNA. The mixture was incubated for 90 min at room temperature in the dark. After ethanol precipitation the pellet was resuspended in 300 μl deprotection buffer (100 mM AcOH adjusted to pH 3.8 with TEMED), heated to 60°C for 30 min (90 min for oligomers containing a biotin modification), again ethanol precipitated and dissolved in 300 μl 100 mM TEAA (pH 7). Purification of labeled oligomers was performed by HPLC on an Äkta Purifier 10 system, using a C8 column (Kromasil 100 C8 7 μm 250 × 4.6 mm) and performing a gradient from 100% TEAA buffer to MeCN/TEAA (50/50) (Supplementary Figure S4). Fractions showing absorbance at 550 nm/650 nm (Cy3/Cy5) and 260 nm were collected, ethanol precipitated and dried. Pellets of fluorophore labeled oligomers were dissolved and pooled in ddH_2_O.

Unlabeled oligomers (10 μl, 1 mM) were deprotected by addition of 90 μl deprotection buffer and incubation for 30 min (90 min for oligomers containing a biotin modification) at 60°C. After ethanol precipitation the RNAs were resuspended in ddH_2_O.

To obtain donor/acceptor or acceptor labeled RNAs, equimolar amounts (2.5 μM) of the respective labeled and unlabeled oligomers and complementary DNA splint in 400 μl 0.5× T4 DNA ligation buffer (NEB) were annealed by heating to 85°C for 5 min and cooling down to room temperature for 15 min. The reaction mixture was adjusted to 1× ligation buffer concentration, 10 μl of T4 DNA ligase (NEB, 2 000 000 units/ml) and 10 μl T4 DNA ligase (ThermoFisher, 5 Weiss U/μl) were added and incubated for 2.5 h at 37°C. Then 10 μl TURBO DNase (Invitrogen, 2 Weiss U/μl) were added and incubated for another 1.5 h to remove the DNA splint. The mixture was phenol extracted, ethanol precipitated and the pellet was dissolved in a mixture of 45 μl ddH_2_O and 45 μl formamide. Fully ligated RNA constructs were separated by PAGE from side products (Supplementary Figure S4), and desired gel bands were excised and extracted in 0.5 M NH_4_OAc at room temperature and 600 rpm for 12 h. Fractions were combined, ethanol precipitated and dried RNA pellets were pooled and resuspended in ddH_2_O.

### smFRET spectroscopy

#### Preparation of cover slips and objective slides for smFRET measurements

Cover slips were cleaned by oxygen plasma for 15 min. Afterwards, silanization was performed with 1% 3-aminopropyltriethoxysilane (v/v) and 5% acetic acid (v/v) in methanol under sonication for 1 min and incubation at room temperature for an additional 20 min. Cover slips were washed with H_2_O, dried with nitrogen and surface functionalization was performed with PEG/PEG-biotin (33 mM mPEG-succinimidyl valerate, MW = 5 kDa; 0.7 mM biotin–PEG–succinimidyl valerate, MW = 5 kDa; NANOCS) in 100 mM bicarbonate buffer at room temperature overnight. A second washing step with H_2_O was performed to remove excess of PEG and cover slips were dried with nitrogen and stored under argon. Objective slides were cleaned prior to smFRET measurement by being exposed to nitrogen plasma for 15 min and measurement channels were generated by combining cover slips and objective slides with double-sided sticky tape.

#### Sample preparation

Reconstitution of RNP complexes was done with 600 nM Cy5-labeled biotinylated snoRNA, 6 μM Cy3-labeled Nhp2 and 6 μM of all other proteins. Samples were incubated at room temperature for 5 min and placed on ice afterwards. Prior to smFRET measurement, each sample was diluted in psi buffer to a final snoRNA concentration of 1 nM. Measurement channels were prepared as described above. Each channel was flushed consecutively with 15 μl of 0.2 mg ml^−1^ streptavidin, 70 μl psi buffer, 10 μl diluted sample and 40 μl psi buffer supplemented with an oxygen-scavenging system (10% glucose (w/w), 14 units ml^−1^ glucose oxidase (Sigma Aldrich), 1000 units ml^−1^ catalase (Sigma Aldrich), Trolox (saturated, Carl Roth)).

#### smFRET measurements and data analysis

SmFRET measurements were performed on an objective-type spinning-spot total internal reflection microscopy setup with an EMCCD camera (iXon, Andor Technology) at 22°C with 532 nm laser (green laser) and 633 nm laser (red laser) excitation with an integration time of 100 ms. For reconstitution experiments, 20 frames with green excitation were recorded for histogram data. Donor only molecules were removed from histograms by fitting a Gaussian distribution around *E*_FRET_ = 0, and subtracting this from the histogram. For experiments with fluorophore labelled Nhp2, 70 frames were collected per movie. Green laser was turned on at the start and red laser was turned on after 25 frames (2.5 s). For histograms, the first 20 frames (2 s) were analyzed and up to 40 movies per sample were analyzed. To only analyze molecules with a FRET pair, the difference between green and red channel in the last 40 frames (4 s) was determined and had to exceed a certain threshold for the molecule to be analyzed (Supplementary Figure S5). For all smFRET experiments, we checked for single-step photobleaching as well as possible FRET dynamics, using single molecule traces generated from 2-min movies (Supplementary Figure S6 and S7). Where applicable, we analyzed time trace data using Hidden Markov model based analysis (HaMMy (version 4.0)) (35).

## RESULTS

### Monitoring RNA conformation during RNP assembly

**Figure 2.**
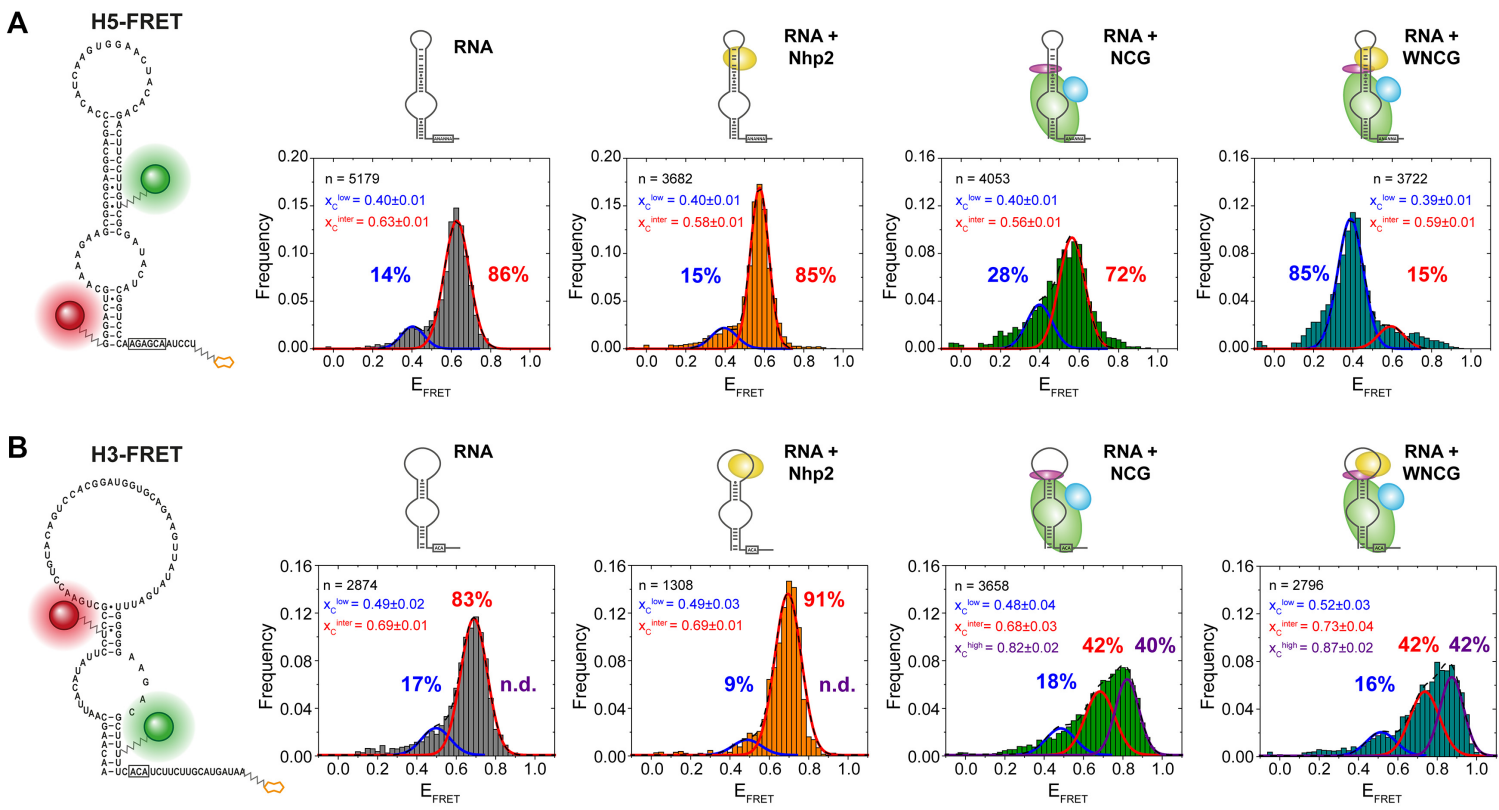
Conformational changes of individual hairpins monitored by EFRET during complex assembly. (**A**) Analysis of H5 assembly, comparing histograms for (from left to right): RNA only, RNA with Nhp2, RNA with Nop10, Cbf5 and Gar1 (‘NCG’), and RNA with NCG and Nhp2 (‘WNCG’). (**B**) Analysis of H3 assembly with identical order of histograms. For RNA constructs, the labeling sites for donor Cy3 (green) and acceptor Cy5 (red) are shown. For histograms, the number of molecules is normalized and plotted as frequency for each data bin. The Gaussian distribution fit parameters are indicated as center of the E_FRET_ distribution x_c_, and fraction of each distribution (indicated in percentages). n indicates the number of molecules used for analysis.

In order to investigate RNA conformation during complex assembly, we performed a stepwise reconstitution of RNPs on individual hairpin RNAs (5′ hairpin H5, and 3′ hairpin H3, see Figure 1). For H5, we placed FRET labels across the pseudouridylation pocket, with the acceptor placed on the 5′ end of the basal helix, and the donor placed on C5 of U61 (Figure 2A). We then reconstituted the RNP complexes, and immobilized them using a biotin handle at the 3′ end of the RNA. Using smFRET, we determined population distributions of the immobilized complexes.

For all H5 complexes analyzed, we could fit two populations (Figure 2A): the first population centered at an *E*_FRET_ = 0.40, and the second one between *E*_FRET_ = 0.56 and 0.63. We term these the low-FRET and the intermediate-FRET population. The difference in FRET efficiency between the two states corresponds to a change in the distance and/or orientation of the two helices towards each other, and due to the position of the labelling sites (Supplementary Figure S8) likely reflects the overall shape and accessibility of the pseudouridylation pocket (Supplementary Figure S9). In the absence of proteins, the dyes are in closer proximity (E_FRET_>0.56), and protein binding then results in the dyes moving further apart, as reflected by the lower FRET efficiency (*E*_FRET_ = 0.40). Both in absence and presence of only Nhp2 protein during reconstitution, the low-FRET conformation was less populated (14% and 15%, respectively). Upon addition of the Nop10-Cbf5-Gar1 (NCG) trimer, this population was increased by about 2-fold (28%). When all four proteins were present, however, the majority of molecules (85%) adopted the low-FRET conformation, showing that this conformational change is caused by a cooperative action of both Nhp2 and the NCG trimer.

For H3, the labeling scheme was slightly altered in order to most faithfully maintain the nucleotide sequence as well as the base pairing pattern of the natural snR81 RNA (Figure 2B). Here, the acceptor in H3 was placed in the apical helix (U21), and the donor in the basal helix (U83). Analogous to H5, a biotin was placed at the 3′ end. For both the H5 and H3 FRET construct, we modelled the accessible volume (36) for each of the four dye attachment sites, using the fully reconstituted archaeal RNP structure (PDB: 3HAY, (25)) as a model. In both cases, this resulted in similar distance vectors spanning across the pseudouridylation pocket (Supplementary Figure S8), rendering the results of H5 and H3 well comparable.

In the H3 construct, three populations were observed, with an *E*_FRET_ ≈ 0.50, *E*_FRET_ ≈ 0.70, and an additional population with an *E*_FRET_ ≈ 0.84, which we here label as low-FRET, intermediate-FRET and high-FRET population, respectively. Given the comparable distance between the fluorophores in each construct (Supplementary Figure S8) and the similar *E*_FRET_ distribution for both H5 and H3 RNAs alone, the population at *E*_FRE__T_ ≈ 0.70 structurally likely corresponds to the intermediate FRET state observed for H5. In contrast to H5, the low-FRET state in H3 does not significantly change with added proteins (17% for RNA alone, and 9–18% for RNPs, Figure 1B). Presence of Nhp2 during reconstitution did not result in a detectable population of molecules adopting the high FRET state. Addition of the NCG trimer resulted in 40% of the molecules shifting into the high-FRET state, which was maintained (at 42%) in presence of all four core proteins.

For the H5 hairpin, we wanted to evaluate whether one of the FRET states observed in the fully assembled construct is indicative of a conformation that may resemble a catalytically active RNP. To this end, we added substrate RNA in excess during the smFRET measurement. Under these conditions, we did not observe a detectable shift in the main low-FRET population centered at *E*_FRET_ = 0.40 (Supplementary Figure S10). In order to verify that in these measurements a detectable amount of RNP complexes has bound a substrate RNA, we reconstituted an RNP where the FRET donor was placed on U61 of H5, and the acceptor on the 3′ end of the target RNA. In this experiment, we indeed detected about 37% of substrate bound RNPs (Supplementary Figure S10).

### Catalytic activity of stand-alone hairpins

We then wanted to test how any of these assembly conformations relate to the catalytic activity conferred by the individual hairpins. For unlabeled RNA, single turnover experiments using site-specifically ^32^P-labeled substrates showed that without the presence of snR81 acting as a guide RNA, the proteins do not exhibit activity on their own (Supplementary Figure S11). In absence of Nop10 or Gar1, again no pseudouridylation was detected. With the addition of H5 or H3 RNAs to the full set of proteins Nhp2, Nop10, Cbf5 and Gar1 (WNCG), both hairpin RNPs showed high enzymatic activity in single turnover assays. When testing the FRET-labeled H5 RNA, it showed only minimally reduced catalytic conversion in single turnover experiments (Supplementary Figure S11).

**Figure 3.**
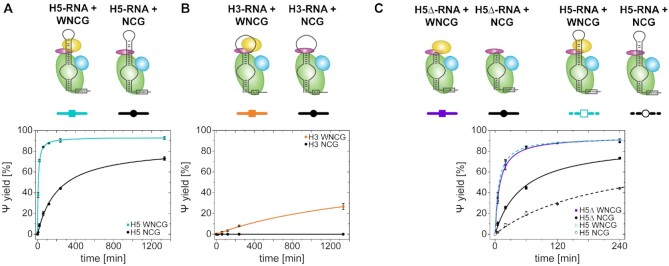
Pseudouridylation activity assays of both snR81 isolated hairpins dependent on the presence of Nhp2. (**A**) Activity of H5 stand-alone hairpin with WNCG (cyan) and NCG (black) complex. (**B**) Activity of H3 stand-alone hairpin with WNCG (orange) and NCG (black) complex. (**C**) Activity of H5Δ versus H5 stand-alone hairpins with WNCG (violet and dashed cyan, respectively) and NCG (black and dashed black, respectively) complex.

For unlabeled H5, the full complex showed almost quantitative turnover of substrate RNA, yielding 93% conversion after 22 h (Figure 3A). In absence of Nhp2, this decreased to 73%. In the case of H3 (Figure 3B), activity is significantly lower, with only 27% conversion after 22 h. Removal of Nhp2 however completely abolishes detectable levels of pseudouridylation.

These results suggest that the structure of the region of the RNA to which Nhp2 binds differs between the two hairpins. In order to investigate the effect of Nhp2 on the apical region of the individual hairpins, we devised truncated constructs for both H5 and H3, termed H5Δ and H3Δ. For H5Δ, H5 was truncated after 5 bp in the apical helix, closed by a UUGG tetraloop, while for H3Δ, H3 was truncated after 5 bp with a GCUU tetraloop.

While for H3Δ, no activity could be observed under any of the conditions tested (data not shown), the truncation of the apical sequence relieved H5Δ partially from its dependence on Nhp2. For the full complex, H5Δ showed virtually the same yield and kinetics as H5 (Figure 3C). In absence of Nhp2 however, the yield for H5Δ strikingly increased to 73% after 4h, compared to 44% after 4h for H5. In H5, the apical RNA motif (together with Cbf5 and Nop10) provides a binding surface for Nhp2. The absence of this RNA motif in H5Δ therefore appears to unexpectedly alleviate the requirement for Nhp2, and allows formation of an active RNP together with NCG, while H5 is more reliant on Nhp2. A control experiment using dye-labeled Nhp2 (Supplementary Figure S12) however shows that Nhp2 does bind to the H5Δ-NCG RNP, showing that the apical RNA binding motif is not strictly required.

To test whether this activity effect correlated with the structural dynamics of the RNA during complex assembly, we devised a FRET construct of H5Δ, analogous to the H5 construct shown in Figure 2. Upon assembly of different RNPs on H5Δ, we found a major FRET state at *E*_FRET_ = 0.60. The population of this state was only minimally affected by addition of either NCG or WNCG (Supplementary Figure S13). Since this state is not shifted even by addition of substrate RNA (Supplementary Figure S10), we assume this state to represent the H5Δ conformation in the catalytically active RNP. We note that the *E*_FRET_ of this state is similar to the intermediate FRET state of H5. However, both the conformational change as well as the catalytic activity of H5 are largely dependent on Nhp2 binding in contrast to the Nhp2-independence of H5Δ. Therefore, the conformation resembling the catalytically active RNP may well exhibit different *E*_FRET_ values for H5 versus H5Δ.

### smFRET analysis of Nhp2 binding to individual hairpin RNPs

**Figure 4.**
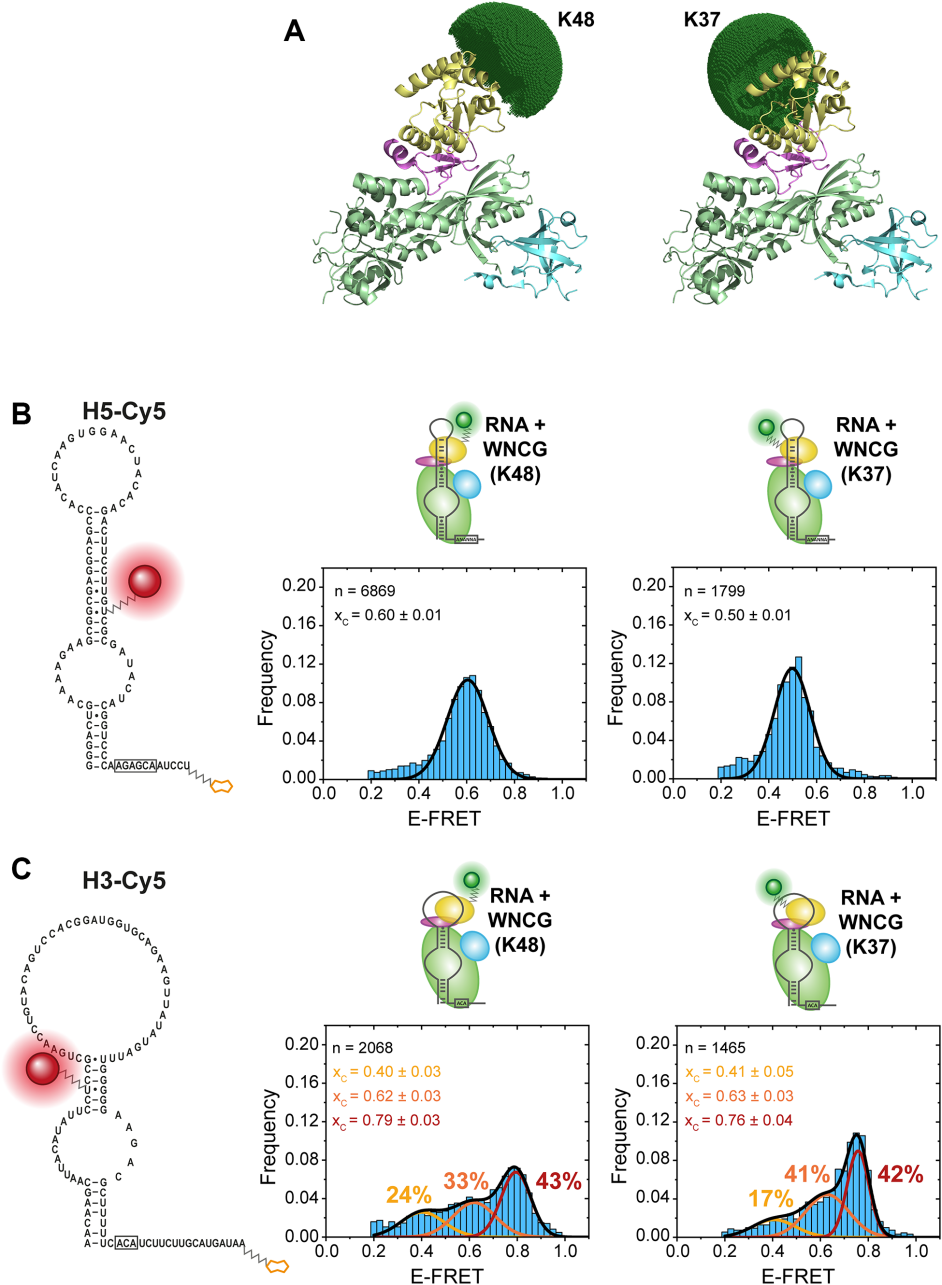
Binding of donor Cy3-labeled Nhp2 constructs K48-Cy3 and K37-Cy3 to the stand-alone hairpins H5 and H3. (**A**) Model structure (PDB: 3U28, 2LBW) with simulated accessible volume for the donor dye. (**B**) smFRET analysis with acceptor-labeled H5 RNA. (**C**) smFRET analysis with acceptor-labeled H3 RNA. For histograms, the number of molecules is normalized and plotted as frequency for each data bin. The Gaussian distribution fit parameters are indicated as center of the *E*_FRET_ distribution *x*_c_, and fraction of each distribution (indicated in percentages). n indicates the number of molecules used for analysis.

The effects of Nhp2 on H5 and H3 RNP assembly prompted us to further investigate how Nhp2 binding to the individual hairpins contributes to folding and activity. To this end, we devised and synthesized fluorophore-labeled Nhp2 in order to assess the binding mode of Nhp2 towards the individual hairpins using smFRET. We placed the acceptor dye in the apical stem labeling sites used for assembly experiments of both the H3 and H5 RNA (Figure 2), and site-specifically introduced the donor dye at positions K37 or K48 of Nhp2 using non-natural amino acid labeling followed by fluorophore coupling (33). In absence of structural data of this RNP, these labeling sites were derived from experiments with the homologous L7Ae (37). We modeled the rotational freedom of the fluorophore using the FPS software package (36) based on a model structure (18,19) (Figure 4A). Labeled Nhp2 protein provided catalytic activity that was close to the level of unlabeled protein (Supplementary Figure S14).

For H5, reconstitution into the full H/ACA RNP with labeled Nhp2 resulted in one predominant distribution at an E_FRET_ = 0.60 with Nhp2-K48Cy3, and 0.50 with Nhp2-K37Cy3 (Figure 4B). In both instances, the corresponding time traces did not show any dynamics on the timescales analyzed (Supplementary Figure S6). This interaction was specific for the fully reconstituted RNP, since omission of either NCG or Nop10 resulted in no detectable binding events (Supplementary Figure S15).

In the case of H3, for each of the protein constructs, three populations were distinguishable (Figure 4C) (with *E*_FRET_ = 0.40, 0.62 and 0.79 for K48, and 0.41, 0.63, and 0.76 for K37, respectively). The individual states were populated to similar degrees for both protein labeling sites. While we could identify time traces for each of the states, these did not show dynamics (i.e. transitions between states) in any case (Supplementary Figure S7). The similar population distribution for both protein constructs suggests that this splitting into three states is likely not caused by artefacts due to protein labeling.

For both H3 and H5 reconstitutions with fluorophore-labeled Nhp2, we wanted to assess whether the conformational distribution responds to the binding of substrate RNA. We repeated the substrate binding experiments described above with both K37 and K48-labeled Nhp2, and again found that none of the reconstituted RNPs showed noticeably altered FRET states in the presence of substrate RNA (Supplementary Figure S16). With no distribution changes in FRET states upon substrate addition, this indicates that the complex already may be in a conformation resembling the catalytically active RNP. Only for the minor exception of acceptor labeled RNA construct H3 and K37-donor-labeled Nhp2, addition of substrate RNA in fact increased the high FRET population. However, this observation is also in line with the FRET and activity data, which indicates that a shift into the high FRET state for H3 is accompanied by an increase in activity (described below).

### Effect of RGG domains on RNP conformation and activity

**Figure 5.**
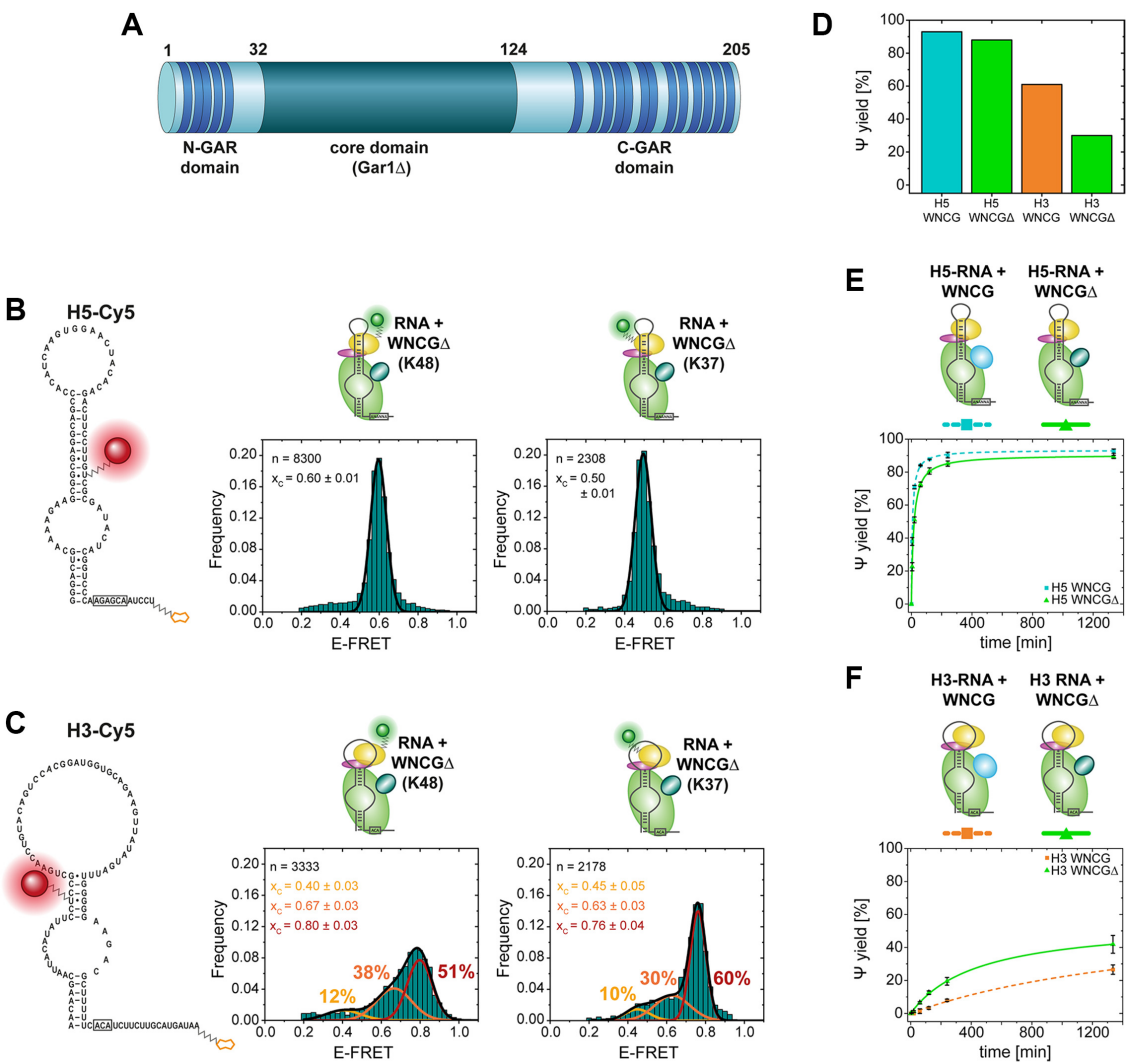
Nhp2 binding to the RNP and catalytic activity in absence of RGG domains of Gar1. (**A**) Linear representation of the amino acid sequence of Gar1, indicating RGG repeats (dark blue). (**B**) smFRET analysis with acceptor-labeled H5 RNA. (**C**) smFRET analysis with acceptor-labeled H3 RNA. (**D**) Final pseudouridylation yield under single-turnover conditions. (**E**) Catalytic activity of RNP with H5 RNA and full-length Gar1 (dashed cyan line) versus Gar1Δ (green line). (**F**) Catalytic activity of RNP with H3 RNA and full-length Gar1 (dashed orange line) versus Gar1Δ (green line). For smFRET histograms, the number of molecules is normalized and plotted as frequency for each data bin. The Gaussian distribution fit parameters are indicated as center of the E_FRET_ distribution *x*_c_, and fraction of each distribution (indicated in percentages). *n* indicates the number of molecules used for analysis.

In the experiments above, the RNA platform for binding of Nhp2 was characterized in presence of all additional eukaryote-specific domains in Cbf5 and Gar1. As Gar1 contains several RGG domains that potentially also bind RNA, we wanted to test their effect on the individual snR81 hairpins. To this end, we prepared a Gar1 construct (Gar1Δ) that, similarly to a truncated construct used before (18), spanned amino acids 32–124 of Gar1, and was devoid of any RGG motifs (Figure 5A).

Using the Nhp2-RNA smFRET approach described above, we found that for H5, the distribution center did not shift [E_FRET_ = 0.60 (K48) and 0.50 (K37)], but determination of the full width at half maximum (FWHM) of the two distributions showed that both became significantly narrower upon removal of the RGG domains [FWHM = 0.15 (K48) and 0.17 (K37) for Gar1 versus FWHM = 0.08 (K48 and K37) for Gar1Δ] (Figure 5B, and Table 1). For the less active H3 in contrast, we did not observe a peak sharpening, but rather a shift of populations in favor of the high-FRET state at *E*_FRET_ ≈ 0.8 for K48 or *E*_FRET_ = 0.76 for K37 (Figure 5C): Here, the low-FRET state (at *E*_FRET_ = 0.40 for K48 or *E*_FRET_ ≈ 0.41 for K37) decreased from 24% to 12% (K48), or from 17% to 10% (K37). The intermediate state (at *E*_FRET_ ≈ 0.62 for K48 or at *E*_FRET_ = 0.63 for K37) shifted from 33% to 38% (K48), or from 41% to 30% (K37). Only the high-FRET state underwent a considerable increase for both protein constructs, from 43% to 51% (K48), or from 42% to 60% (K37). In either case, the distribution width did not change significantly (Table 1).

**Table 1.** Comparison of FRET efficiency distribution center (*x*_c_) and distribution width (FWHM) of the high-FRET state of acceptor-labeled H5 and H3 upon binding to donor-labeled Nhp2

	H5 K48	H5 K37	H3 K48 (high-FRET state)	H3 K37 (high-FRET state)
*E*_FRET_ *x*_c_ with Gar1	0.60 ± 0.01	0.50 ± 0.01	0.79 ± 0.03	0.76 ± 0.04
*E*_FRET_ *x*_c_ with Gar1Δ	0.60 ± 0.01	0.50 ± 0.01	0.80 ± 0.03	0.76 ± 0.04
FWHM with Gar1	0.17 ± 0.01	0.15 ± 0.01	0.12 ± 0.02	0.09 ± 0.01
FWHM with Gar1Δ	0.08 ± 0.01	0.08 ± 0.01	0.13 ± 0.02	0.08 ± 0.01

In correlation with the activity data (Figure 5D), this might point toward the high FRET state (*E*_FRET_ = 0.76–0.80) representing an Nhp2-bound conformation that may resemble a catalytically active RNP. Since only roughly half of the observed molecules are forming this suggestedly functional conformation of the RNP, this again might be a partial explanation for the overall lower activity of the 3′ hairpin.

For all of these conditions, we again assessed the effect of substrate RNA on the FRET distributions. Consistent with the experiments described above, none of the constructs showed a pronounced shift in FRET states in presence of substrate RNA (Supplementary Figure S16). We then tested whether the presence of the RGG domains had an impact on the catalytic activity for each hairpin under multiple turnover conditions. For H5, the activity was slightly impeded by deletion of the RGG domains (Figure 5E), with 89% final yield and 1.76 min^−1^ turnover rate for Gar1Δ instead of 93% yield and 3.04 min^−1^ turnover rate for the full-length Gar1. In contrast, the activity of H3 was markedly increased (42% yield in Gar1Δ versus 27% for Gar1) (Figure 5F). As archaeal Gar1 has an impact on both single- und multiple turnover catalysis (14,17,25,38), we also tested the activity with an excess RNP over substrate, and found that the pseudouridylation yield for H5 was decreased from 93% to 88%, consistent with multiple turnover experiments. For H3 however, the yield was in fact decreased from 61% to 30% (Figure 5D), which contrasts the roughly 2-fold yield increase detected under multiple turnover conditions, and the five times faster initial turnover rate (H3 Gar1: 0,01 min^−1^, H3 Gar1Δ: 0.05 min^−1^) (for an overview of all turnover rates discussed below, see Supplementary Table S4).

### Activity in the context of eukaryote-specific features in the full snR81 RNP

When testing similar conditions for snR5 and snR34, previous studies found that the bipartite architecture of eukaryotic H/ACA RNPs, more precisely the presence of both hairpins, can affect catalytic activity (18,24). The fact that both the exchange of L7Ae against Nhp2, the occurrence of RGG domains in Gar1, and the bipartite architecture appear to be evolutionarily linked in eukaryotes prompted us to investigate the effects of these characteristics and compare them against each other.

**Figure 6.**
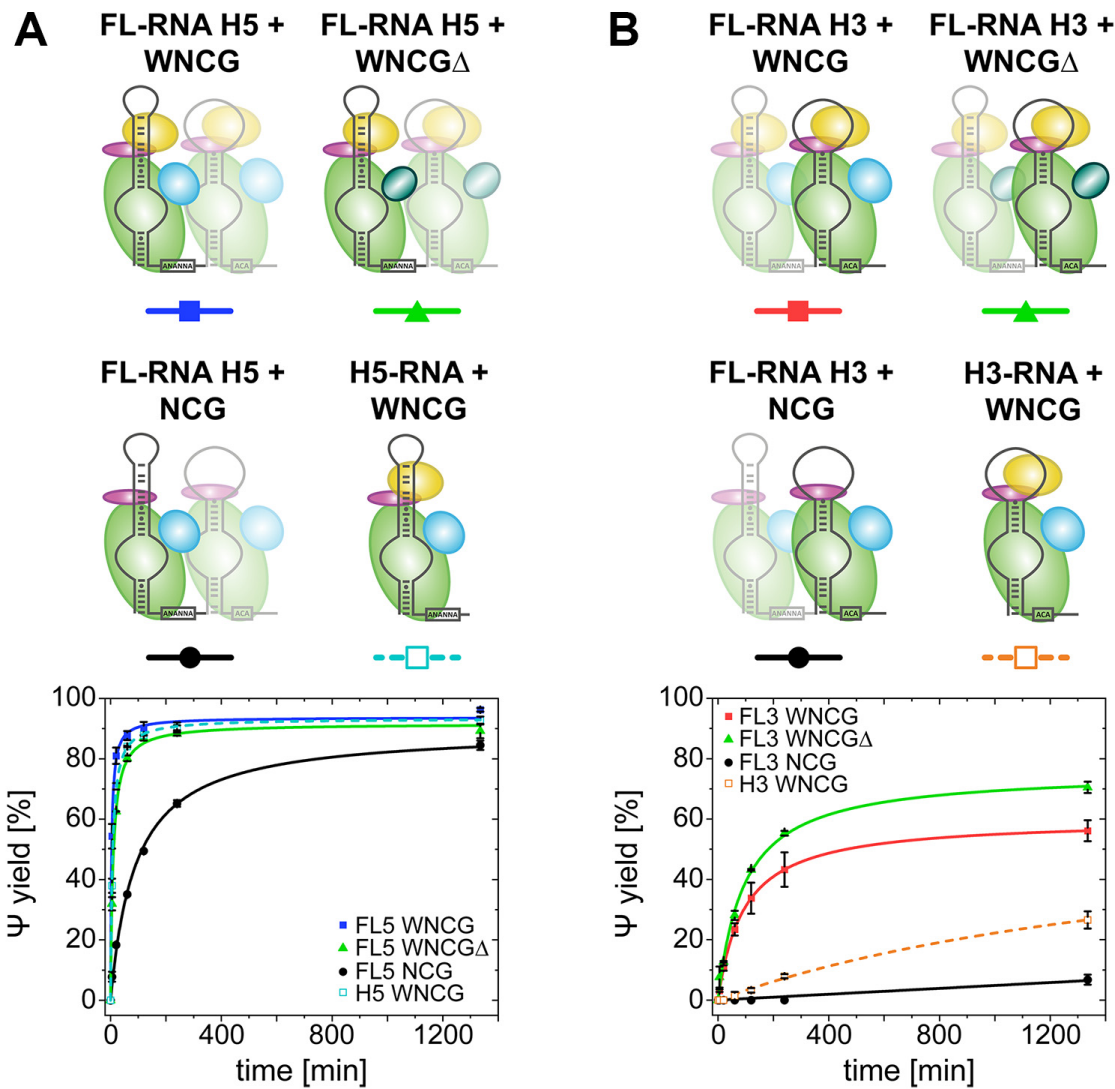
Catalytic activity of individual hairpin RNAs in context of the full-length snR81 sequence. (**A**) Pseudouridylation activity for H5 in the full snR81 RNP (blue line), with Gar1Δ (green line), without Nhp2 (black line). Standalone hairpin H5 for comparison (dashed cyan line). (**B**) Pseudouridylation activity for H3 in the full snR81 RNP (red line), with Gar1Δ (green line), without Nhp2 (black line). Standalone hairpin H3 for comparison (dashed orange line).

When testing the activity of single hairpin RNAs in fully assembled complexes versus the full-length snR81, we found that for H5, the starting turnover rate was indeed increased by about 38% for snR81 (4.21 min^−1^ versus 3.04 min^−1^), and the final yield was slightly increased from 93% to 96% after 22 h (Figure 6A). For H3, the stabilizing effect across hairpins was more pronounced, with an increase from 0.01min^-1^ initial turnover rate in H3 alone to 0.24 min^−1^ in snR81, and an increase from 27% to 56%, respectively, in final yield after 22h (Figure 6B).

To test whether Nhp2 or RGG domains had an impact in a setting where both hairpins are present and functional, we repeated these experiments, omitting each (Nhp2 protein or RGG domains) individually. When removing Nhp2, the activity of H5 in the full-length snR81 was decreased in both starting turnover rate (0.15 min^−1^ for H5 versus 0.37 min^−1^ for snR81) and yield (73% versus 85% after 22 h). In absence of Nhp2, H3 in the full-length snR81 yielded low levels of pseudouridylation (7% after 22h), in contrast to the virtually inactive H3 alone.

Deletion of the RGG domains had a consistent effect on the activity of H5 in the full-length (FL) snR81, as it resulted in an only minor decrease in activity (4.21 min^−1^ for Gar1 versus 2.53 min^−1^ for Gar1Δ in snR81), but had no discernible effect on the yield (89%).

In the case of H3 activity in full-length snR81, the effect of RGG domains was again consistent with the standalone hairpin, as RGG domain removal resulted in increased activity (0.31 min^−1^ versus 0.24 min^−1^) and higher yield (70% versus 56%).

## DISCUSSION

### Assembly depends on the protein backbone trimer Cbf5-Nop10-Nhp2 altering RNA conformation

During assembly of the H/ACA RNP, the protein trimer Cbf5-Nop10-Nhp2 forms a platform for cotranscriptional binding of a single snoRNA hairpin. Only upon cellular localization, Gar1 is binding to the complex, rendering it catalytically active. In this regard, it is interesting to see that binding of either Nhp2 alone or Cbf5-Nop10 (with Gar1) have limited effects on the conformation of the 5′ hairpin of snR81 (28% in low FRET population). Only the combination of all proteins results in a near quantitative (83%) shift into a different fold in our smFRET experiments, suggesting that the RNA is adopting a state that may well resemble the catalytically active conformation. Complex formation on H5 appears to result in a widening conformational change of the pseudouridylation pocket, and we therefore assume the low FRET state (*E*_FRET_ = 0.40) to represent an ‘open’ conformation, and the intermediate FRET state (*E*_FRET_ > 0.56) to be indicative of a ‘closed’ conformation (Supplementary Figure S9). For H5, these findings are therefore in line with the Cbf5-Nop10-Nhp2 trimer forming the backbone of the RNP (18,39). In fact, the data from both assembly experiments (Figure 2), activity data in absence of Nop10 (Supplementary Figure S11), and the lack of Nhp2 binding events in absence of Nop10 (Supplementary Figure S15) highlight the essential role Nop10 plays in correctly assembling a catalytically active RNP complex. For the 3′ hairpin, this effect is however different, with the refolding into a high FRET state observable with NCG already in absence of Nhp2 (40%, and 42% with Nhp2). In these experiments, this may be due to a weaker binding of Nhp2 to the 3′ hairpin, or partial misfolding of the RNA, even within the NCG-bound RNP. Limited binding of Nhp2 to a subset of correctly folded H3 molecules would also explain the abundance of only a limited number of molecules in the high FRET state. Due to the comparable labeling scheme (Supplementary Figure S8), the conformational change of H5 and H3 upon RNP formation likely occurs in opposite directions. At the same time, some molecules adopt a low-FRET state. Since this state however is already present in absence of proteins, and does not increase upon RNP formation, there is a possibility that this state originates from misfolded RNAs. We therefore cannot assign one of the three FRET states of H3 to be representative of the active conformation in these assembly experiments. Instead, this differential behaviour between H3 and H5 may be linked to distinct features that are unique for each hairpin (such as the basepairing in the pseudouridylation pocket, or the degree of structure in the apical stem/loop structure) in this or other snoRNAs.

In the 5′ hairpin of snR81, the degree of structure in the apical part above the pseudouridylation pocket is generally high. The long helical region might contribute to the correct folding in absence of Nhp2, and binding of Nhp2 would then stabilize this pre-existing secondary structure of the upper stem, and anchor it on the RNP. This anchoring increases catalytic activity, and results in a conformational change observed in our FRET experiments. For H5Δ, truncating the apical part of the RNA results in a prefolded RNA conformation, which alleviates the requirement for Nhp2 for both, anchoring the RNA and catalytic activity.

In comparison to the 5′ hairpin, the 3′ hairpin features fewer basepairs in the apical stem above the pseudouridylation pocket with a larger, unstructured loop region. Here, stabilization of the correct folding in this part of the RNA as well as anchoring onto the RNP, both mediated by Nhp2, are strictly required for activity. This anchoring may be even more important than in H5, as truncation of the RNA leaving only a short apical hairpin structure (H3Δ) renders the RNA catalytically inactive. Overall, folding of the apical part of either RNA hairpin may therefore be one of the limiting factors for reconstituting catalytically active RNPs.

Regarding the interaction between Nhp2 and the apical parts of the snoRNA hairpins, it is interesting to investigate how Nhp2 binds to either of the hairpins. To this end, we successfully prepared two site-specifically fluorophore labeled Nhp2 constructs, and were able to characterize their binding to each snoRNA hairpin on the level of individual molecules. For H5, the distinct peaks for both protein labeling sites suggest that there is indeed a predominant, well-defined binding mode of Nhp2 in the context of the full RNP. For H3 however, the three distinct FRET states presumably correspond to three distinct binding modes of Nhp2. While the high-FRET state can be assumed as the active conformation (which in the case of K37-labeled Nhp2 binding to the full RNP may be increased upon binding of substrate RNA), the two populations with lower *E*_FRET_ can be attributed either to partially assembled complexes, or to two distinct conformations within (fully or incompletely) reconstituted complexes due to partial RNA misfolding.

In summary, Nhp2 likely stabilizes the active conformation of each snoRNA hairpin despite its rather promiscuous binding of RNA structural elements (40,41). Together with the anchoring functionality that was described in this work as well as previous studies (24,39,42), it contributes to the overall folding and activity of the H/ACA RNP. The finding that the major conformation of each hairpin is not altered upon substrate RNA binding strongly suggests that these indeed represent the active conformations. The diverging effects Nhp2 binding has on the apical RNA parts are likely due to differences in sequence and/or the degree of structure.

### RGG domains affect stability of the RNP, and may impact RNP-substrate interaction

The observed instances of misfolding in H3 may be due to additional interactions between the RNA and other protein domains. We therefore investigated the effect of RGG domains in Gar1 on both RNP conformation and activity. It currently is unclear whether these eukaryote-specific domains interact with either substrate RNAs, snoRNAs within the same RNP, or other cellular RNAs that are i.e. involved in liquid-liquid phase separation (32). For Nhp2 binding to H5 (Figure 5), we found that removal of the RGG domains decreased the population distribution width, indicating less movement or fewer dynamics within the complex. Since unbiased HaMMy analysis did not yield discernible folding transitions, this suggests a more rigid structure. In turn, this would indicate that interactions of the RGG domains with other parts of the RNP complex indeed decrease at least structural rigidity in these experiments. For H3, the distribution width of all populations (and for both protein labeling sites) remained unchanged, but instead the molecules were shifted into the population with the highest FRET efficiency (*E*_FRET_ ≈ 0.8). Assuming that this state corresponds to the catalytically active fold, the RGG domains would aid in folding the RNA into this conformation.

For H5, removal of the RGG domains has a minor inhibitory effect on the high level of pseudouridylation in both single and multiple turnover experiments. H3 also shows a decrease under single turnover conditions, but unexpectedly an increase under multiple turnover conditions in absence of the RGG domains. This strongly suggests that the RGG domains facilitate catalysis of bound RNA substrates in the 3′ hairpin, but interfere with substrate release and/or exchange of product against new substrate molecules. This points towards an interaction between the RGG domains and some part of the substrate RNA, similar to an effect described for the RGG domain in the METTL3/METTL14 methyltransferase complex (43).

### Substrate binding and functional differences between snoRNAs

Regarding interactions between the substrate RNA and the modifying RNP, it was proposed that the RNA-RNA interactions affect the corresponding turnover rate (18,44). With the 5′ substrate forming 11 A–U and three G–C basepairs with the guide RNA, and the 3′ substrate only forming 10 A–U and 2 G–C basepairs, our results at face value are not in agreement with the study by Li *et al.* (18), where a lower interaction strength would correlate with higher activity. They would however conform with a model proposing higher pseudouridylation kinetics with more stable interactions (44), especially regarding the symmetry of the interactions with parts of the substrate RNA located 3′ and 5′ of the pseudouridylation site. Along the same line, data derived from *ex vivo* isolated snR81 H/ACA RNPs (45) shows that 8 bp may be required, with at least 3 bp in either side of the pocket for efficient modification. Generalization of such findings is further complicated by two additional factors: first, different hairpins may modify not only different target sequences, but entirely different RNA species, concomitant with second, a different subcellular localization. This also holds true for snR81 investigated here (H3: 25S rRNA in the nucleolus, and H5: U2 snRNA in Cajal bodies), and is further emphasized by the report of stress-induced near-cognate modification events (46), which is generally in agreement with the reported base pairing requirements (45). There is however a lack of knowledge on correlation between, i.e. potential structural or sequence features and the localization of any given snoRNA or scaRNA (beyond the CAB-box), and potential proteins that would recognize such features. Taken together, these to some extent divergent findings for different snoRNAs emphasize the need for further research into the exact activity of individual snoRNAs and their RNPs.

### Eukaryote-specific bipartite architecture enhances activity of the RNP

In full-length snoRNA snR81, we find that the effects of the presence of the second hairpin RNP vary between H3 and H5. Activity is overall enhanced for both hairpins, in line with previously published data (18,24). The effect under multiple turnover conditions on H5 is rather limited, while for H3 the initial turnover is increased >20-fold, and the yield after 22 h more than twofold. This suggests that the presence of the second hairpin H5 and/or the proteins assembled on H5 aids in forming a more active complex especially on the 3′ hairpin. This may be due to stabilization of the RNP (i.e. by interactions between proteins and/or snR81), or by inducing a more active RNA fold.

### Eukaryote-specific protein features do not contribute to functional interactions between individual hairpins within the full-length snR81 RNP

To identify potential effects of protein features across hairpins, we compared the impact of Nhp2 and RGG domains of Gar1 on activities of the individual hairpins versus the FL snR81.

From H5 to snR81 in the fully assembled complex, activity increases by 38%. In absence of Nhp2, this effect is however more pronounced (+147%). This suggests a stabilizing influence of the H3 RNP on H5, while Nhp2 may unexpectedly interfere with this stabilizing effect across hairpins. For H3, presence of the H5 RNP leads to a very low level of activity after 22 h in the absence of Nhp2, suggesting a limited stabilizing effect, which is difficult to quantify in our experiments.

Upon removal of the RGG domains, the activity enhancing effect from H5 to snR81 is at 44% (similar to the 38% in the fully assembled complex). For the 3′ hairpin, the activity increase from H3 to snR81 with full-length Gar1 (∼25-fold faster turnover rate, and 2-fold yield increase) is largely maintained for Gar1Δ, with a sixfold increase in initial turnover rate, and almost twofold increase in yield (70% versus 42% after 22 h). These findings may point toward an effect of RGG domains that is limited to the hairpin that Gar1 is associated to, and virtually no effect across hairpins.

In summary, the effects of RGG domains (activity increase for H5 and decrease for H3) as well as Nhp2 (activity increase for H5 and H3) are in general consistent between the stand-alone hairpins and the full-length snR81 complex. This suggests that the activity increase in the context of the full-length snR81 RNP for each hairpin likely is not mediated by interactions between either Nhp2 or RGG domains and the other hairpin RNP. Instead, it suggests that activity-enhancing cross-hairpin interactions occur between either other protein domains (i.e. Cbf5 or Gar1 core domain), the two RNA hairpins, or a protein domain (i.e. Cbf5 or Gar1 core domain) and the respective other RNA hairpin. These interaction(s) then may increase RNP stability or enhance RNA folding, resulting in higher activity.

Our model therefore may be compatible with the model structure proposed from the H/ACA components of human telomerase (20), but likely rules out Nhp2 or the RGG domains of Gar1 as mediators for interactions between the two hairpins.

## Supplementary Material

gkab177_Supplemental_File
