# Supplementary material for: Eukaryote specific RNA and protein features facilitate assembly and catalysis of H/ACA snoRNPs
Source: Nucleic Acids Res. 2021 Apr 6;49(8):4629–42. doi: 10.1093/nar/gkab177 (PMC8096250; doi:10.1093/nar/gkab177)
Supplement: gkab177_Supplemental_File [file gkab177_supplemental_file.pdf]

Supplementary Material

Eukaryote specific RNA and protein features facilitate assembly and catalysis of H/ACA snoRNPs

Sven Trucks, Gerd Hanspach and Martin Hengesbach

Table S1: DNA templates for snoRNA transcription, PCR primers for template generation and mutagenesis primers.

**Transcription templates**	
H5	TAATACGACTCACTATAGGGACTGCAAAAGAAGCGGCGAGGCAGCCCACATCAAGTGGAACTACACAGACTTCCTTGTCGCGATACTACGGTCCCAAGAGCAATCCT
H5Δ	TAATACGACTCACTATAGGGACUGCAAAAGAAGCGGCUUGGGUCGCGAUACUACGGUCCCAAGAGCAAUCCU
H3	TAATACGACTCACTATAGGGAACAAGCAATTACATATTCCCCCGCTGAACCTGTACAGTCCACGGATGGTGCAGAAGTTATATGATTTGGGGGAAGACGCTTTTTCACATCTTCTTGCATGATAA
H3Δ	TAATACGACTCACTATAGGGAACAAGCAATTACATATTCCCCCGCTTGGGGGAAGACGCTTTTTCACATCTTCTTGCATGATAA
FL	TAATACGACTCACTATAGGGACTGCAAAAGAAGCGGCGAGGCAGCCCACATCAAGTGGAACTACACAGACTTCCTTGTCGCGATACTACGGTCCCAAGAGCAATCCTAACAAGCAATTACATATTCCCCCGCTGAACCTGTACAGTCCACGGATGGTGCAGAAGTTATATGATTTGGGGGAAGACGCTTTTTCACATCTTCTTGCATGATAA
**Template primers**	
H5 fwd	TAATACGACTCACTATAGGG
H5 rev	AGGATTGCTCTTGGGACC
H3 fwd	TAATACGACTCACTATAGGGAACAAGCAATTACATATTCCC
H3 rev	TTATCATGCAAGAAGATGTG
**Mutagenesis primers**	
Nhp2 S82W fwd	GTCATTGCCGGTGATATCTGGCCAGCTGACGTTATCTCG
Nhp2 S82W rev	CGAGATAACGTCAGCTGGCCAGATATCACCGGCAATGAC
Nhp2 K48X fwd	AAGTCCTCAAAACCGTGTAGAAAGCGAGTAAAGCCAA
Nhp2 K48X rev	TTGGCTTTACTCGCTTTCTACACGGTTTTGAGGACTT
Nhp2 K37X fwd	GCGAAACCTCTGGCGAGCTAGAAACTGAACAAAAAAGTC
Nhp2 K37X rev	GACTTTTTTGTTCAGTTTCTAGCTCGCCAGAGGTTTCGC

Table S2: RNA ligation fragments and DNA splints for substrate RNA synthesis. The target uridine is labeled with an asterisk.

S5-1	3’ RNA fragment	*UGUUCUUUUCAG
S5-2	5’ RNA fragment	GGGAGUAGUAUC
S5-S	DNA splint	CTGAAAAGAACAGATACTACTCCC
S3-1	3’ RNA fragment	*UAAAUAUGUAAGAA
S3-2	5’ RNA fragment	GGGAACUU
S3-S	DNA splint	TTCTTACATATTTAAAGTTCCC

Table S3: RNA ligation fragments for fluorophore labeled snoRNA. Capital letters mark amino modified nucleotides ("5'-Aminohexyl modifier C6" for N6, "5'-Aminoallyl-uridine" for 5-N and "5'-Aminohexylacrylamino-uridine" for 5-LC-N). Phosphate groups for enzymatic ligation are marked as p, and a biotin linker "3-Biotin" is attached at the 3'-end for immobilization of the complex.

H5/H5Δ 5' fragment	(**N6-G**)GG ACU GCA AAA GAA GCG G
H5 middle fragment	pCG AGG CAG CCC ACA UCA AGU GGA ACU ACA CAG ACU UCC UUG (**5-N-U**)CG C
H5Δ middle fragment	pCU UCG G(**5-N-U**)C GC
H5/H5Δ 3' fragment	pGA UAC UAC GGU CCC AAG AGC AAU CCU-biotin
H3 5' fragment	pAAC AAG CAA UUA CAU AUU CC(**5-LC-N-U**) CCG CU
H3 middle fragment	pGAA CCU GUA CAG UCC ACG GAU GGU GCA GAA GUU AUA UGA UU
H3 3' fragment	pU GGG GGA AGA CGC UU(**5-LC-N-U**) UUC ACA UCU UCU UGC AUG AUA A-biotin

Table S4: Starting turnover rates v_{start} for all snoRNP constructs during the first 5 minutes of the pseudouridylation reaction. Starting turnover rates were determined via linear regression.

v_{start} [min^{-1}]	**FL5**	**H5**	**H5Δ**	**FL3**	**H3**	**H3Δ**
**WNCG**	4.21	3.04	2.67	0.24	0.01	n.d.
**WNCGΔ**	2.53	1.76	0.58	0.31	0.05	n.d.
**NCG**	0.37	0.15	0.61	n.d.	n.d.	n.d.

Supplementary Figure S1

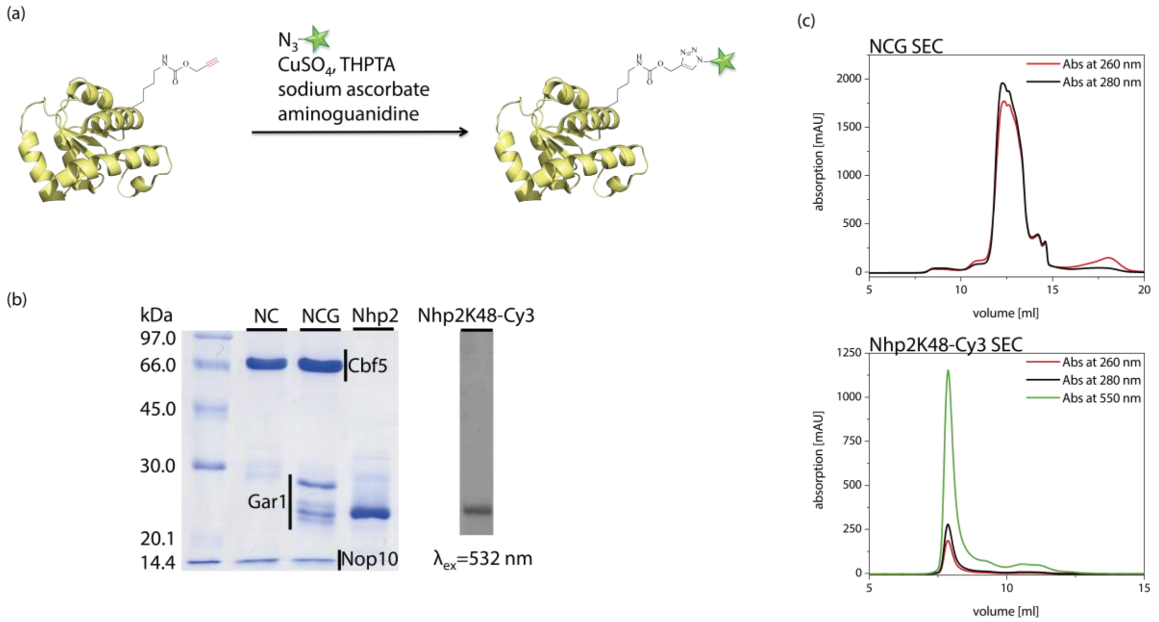

Figure S1: (a) Schematic representation of labeling reaction of Nhp2K48PrK with Sulfo-Cy3-azide. (b) Gel analysis of purified proteins, stained with Coomassie blue and under fluorescence. (c) Chromatograms of SEC for NCG and Nhp2WK48-Cy3.

Supplementary Figure S2

a)

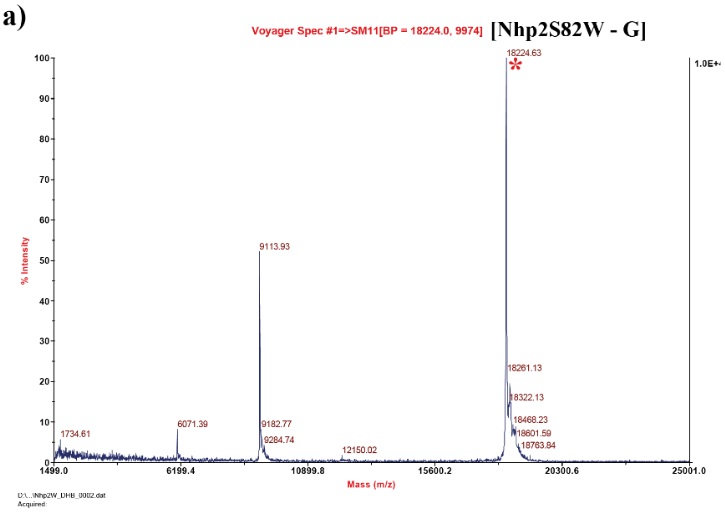

b)

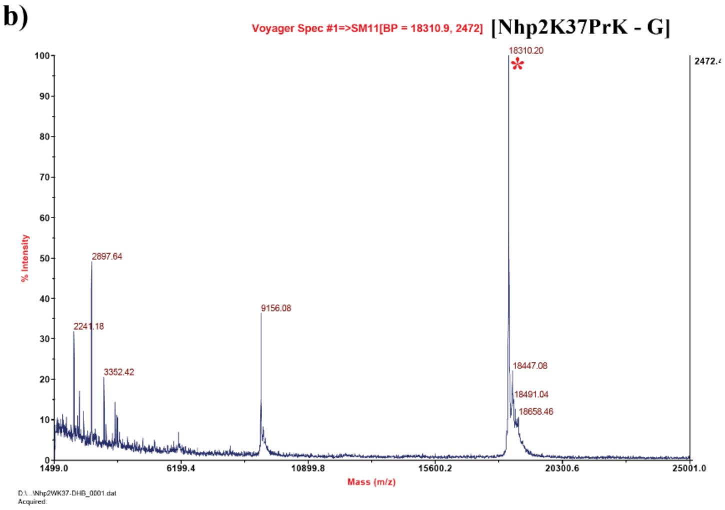

c)

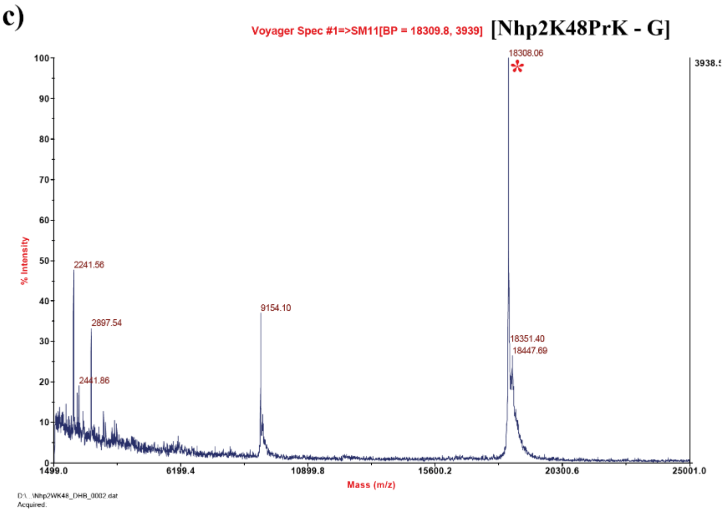

Figure S2: MALDI data of Nhp2S82W (W) (a), Nhp2K37PrK (b), and Nhp2K48PrK (c).

Supplementary Figure S3

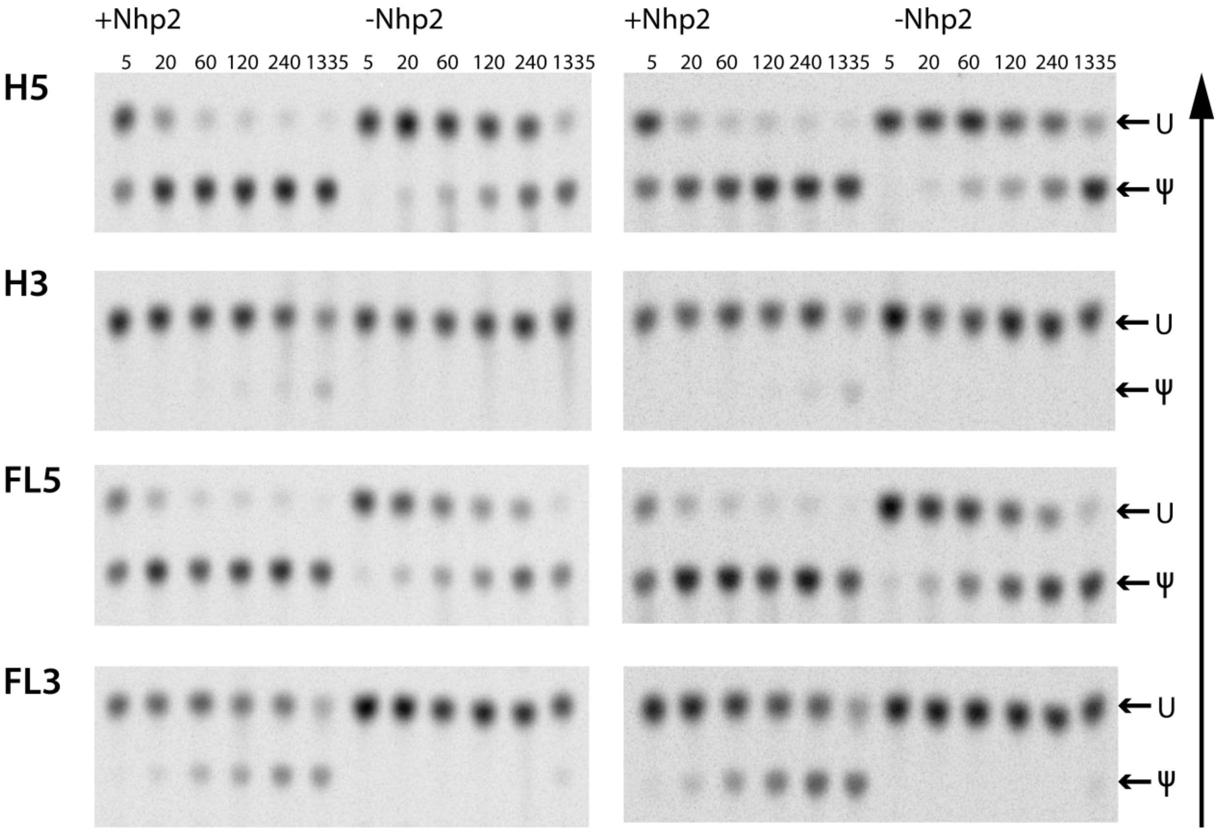

Figure S3: Thin layer chromatography of multiple turnover pseudouridylation assays in presence and absence of Nhp2 (numbers indicate timepoint of sample-taking in minutes).

Supplementary Figure S4

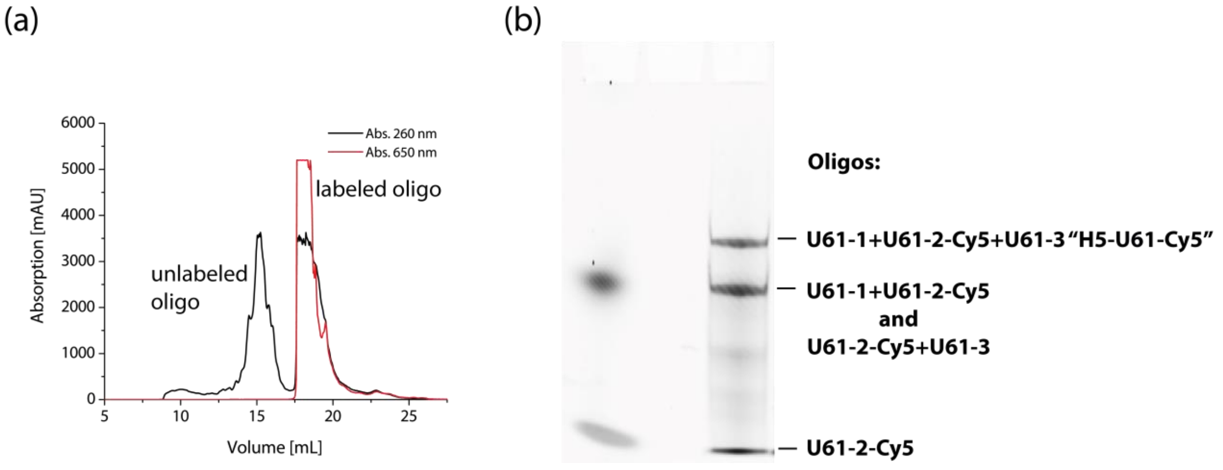

Figure S4: Exemplary purification of Cy5-labeled RNA; H5 U61 construct (U61-1, U61-2 and U61-3 represent the 5', middle and 3' fragments, respectively). (a) HPLC purification of U61-2-Cy5. (b) 8% PAA gel (λ_{es}=633 nm) of ligation products.

Supplementary Figure S5

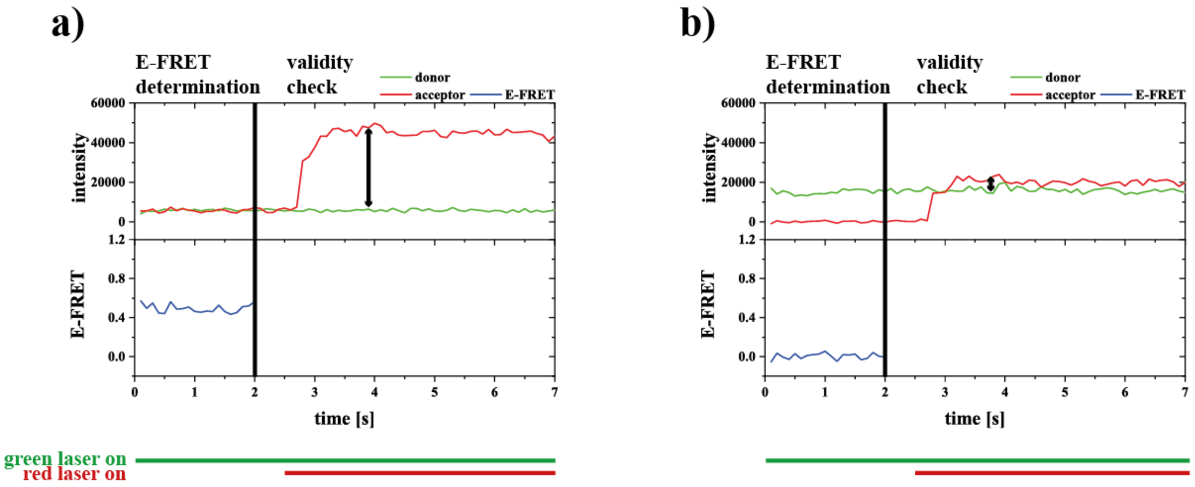

Figure S5: Schematic representation of acceptor filtered smFRET movies. In the first 2 seconds of the measurement, only the green laser is turned on. E_{FRET} values are determined using this initial step via the equation:

$$E_{FRET} = \frac{intensity_{acc}}{intensity_{don} + intensity_{acc}}.$$

Between second 2 to 3, the red laser is manually turned on in addition to the green laser, and remains on until the end of the movie. The mean value between 3.5 and 7 seconds is used for donor and acceptor intensities. The difference between the donor and acceptor values are calculated. Only those traces are accepted for histograms where the difference between donor and acceptor exceeds a set threshold (a). If this threshold is not exceeded, the acceptor dye is considered not to be present and the increase in acceptor intensity arises from the background intensity of the red laser (b). Hence, the trace is discarded and the corresponding E_{FRET} value is not used for the histograms. With this approach, molecules that solely contribute to a donor-only peak are filtered out of the analysis.

Supplementary Figure S6

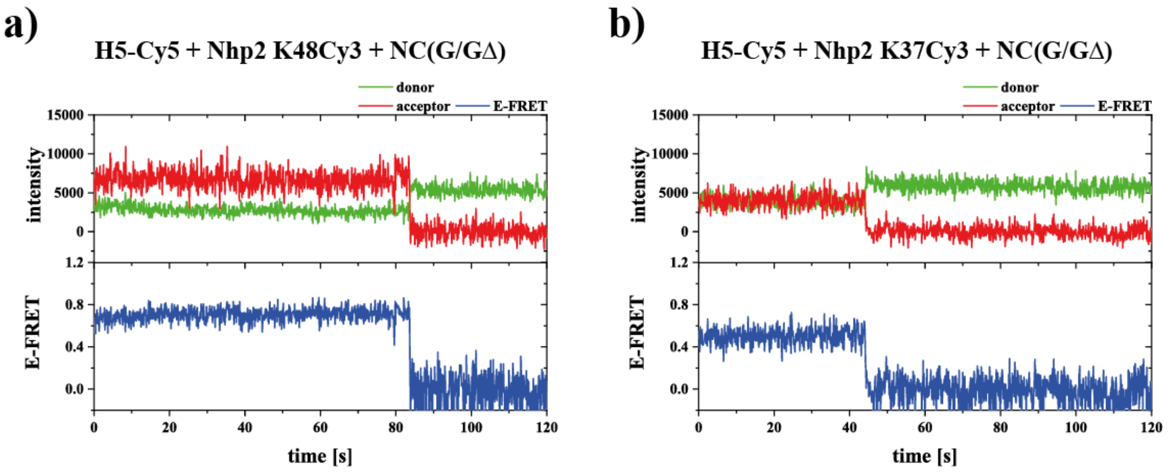

Figure S6: Representative 2-minute smFRET movies for H5-Cy5 and Nhp2 K48Cy3 (a) and Nhp2 K37Cy3 (b). These movies were recorded to observe possible dynamic events occurring in the period of 2 minutes, e.g. a change in E_{FRET} values due to a change in conformation of the complex between both labels. No such dynamic events were observable. The only visible events are donor bleaching as well as donor and acceptor bleaching. These events happen due to a prolonged exposition of the dye molecules to the green laser, resulting in either the donor molecule bleaching first, in which case donor and acceptor intensities drop to 0, or the acceptor molecule bleaching first, in which case the acceptor intensity drops to 0, while the donor intensity shows anti-correlated behavior. After some time, the donor molecule also bleaches, which drops the donor intensity to 0.

Supplementary Figure S7

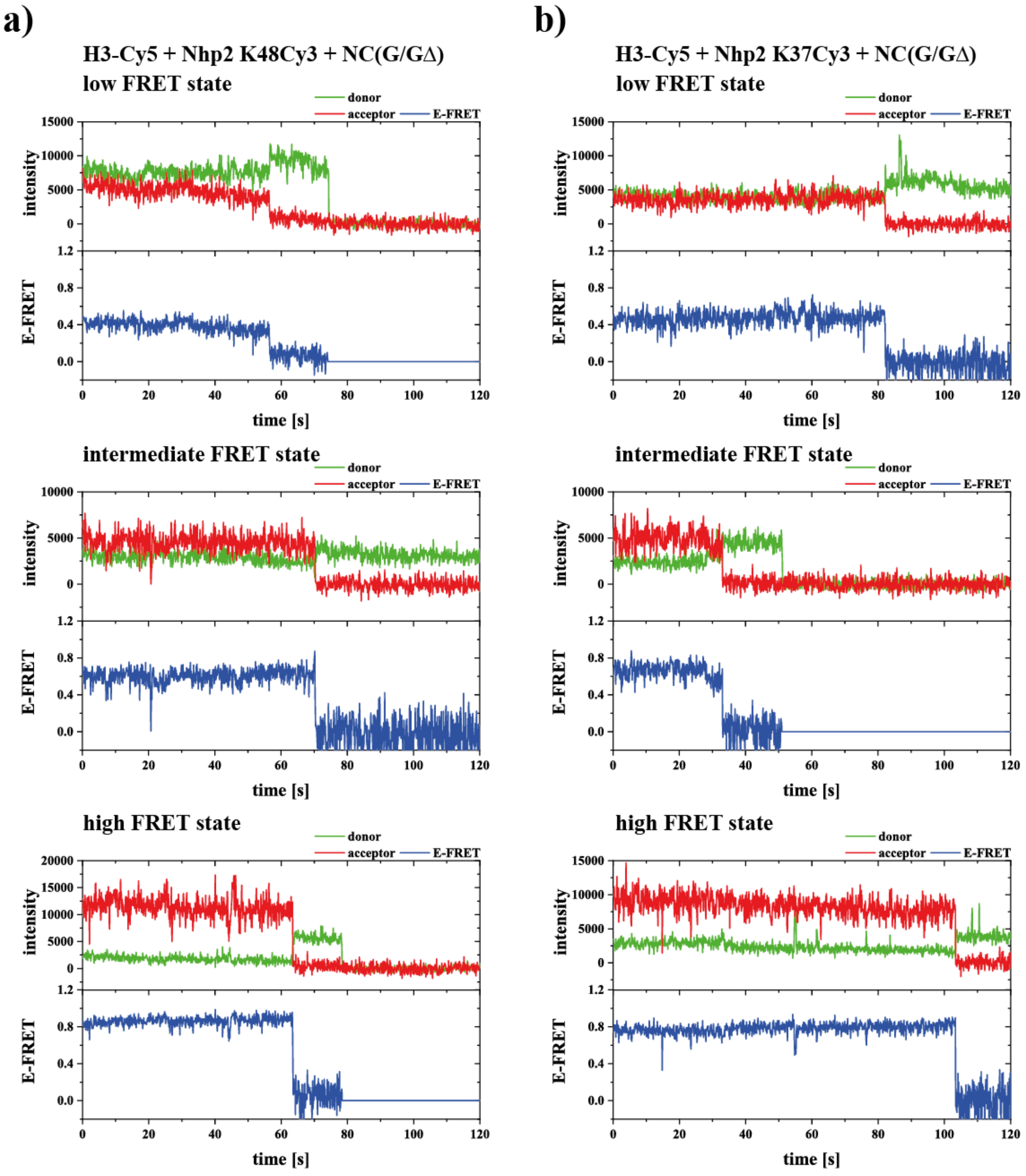

Figure S7: Representative 2-minute smFRET movies for H3-Cy5 and Nhp2 K48Cy3 (a) and Nhp2 K37Cy3 (b). These movies were recorded to observe possible dynamic events occurring in the period of 2 minutes, e.g. a change in E_{FRET} values due to a change in conformation of the complex between both labels. No such dynamic events were observable. The only visible events are donor bleaching as well as donor and acceptor bleaching. These events happen due to a prolonged exposition of the dye molecules to the green laser, resulting in either the donor molecule bleaching first, in which case donor and acceptor intensities drop to 0, or the acceptor molecule bleaching first, in which case the acceptor intensity drops to 0, while the donor intensity shows anti-correlated behavior. After some time, the donor molecule also bleaches, which drops the donor intensity to 0.

Supplementary Figure S8

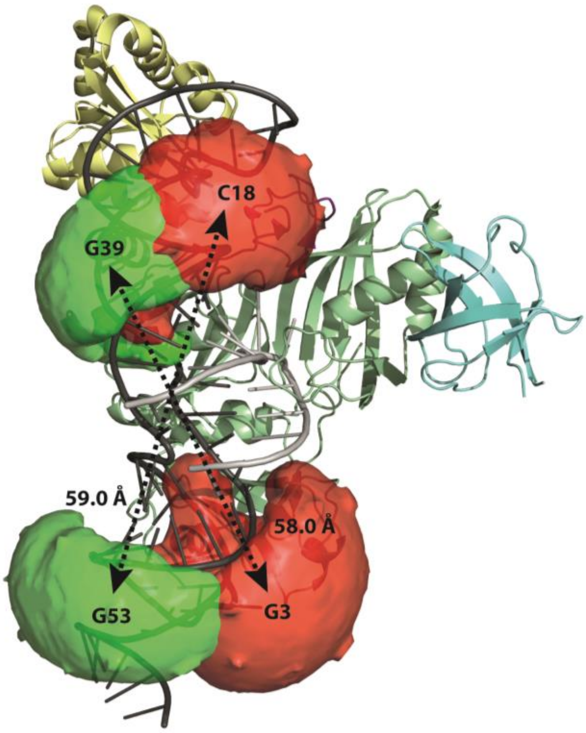

Figure S8: FRET constructs for H5 and H3: FPS modeling of accessible volume clouds for Cy3 (green) and Cy5 (red). Modeling was performed using the archaeal full RNP structure (PDB: 3HAY) and the nucleotides used for modelling dye placement were chosen by overlaying the secondary structures of the archaeal RNA with snR81. Numbering in the picture is according to the archaeal nucleotides. Distances were derived from <RDA> in the FPS modeling software and showed 58.0 Å for the H5 RNA and 59.0 Å for the H3 RNA. FPS modeling was done as previously described (1,2).

Supplementary Figure S9

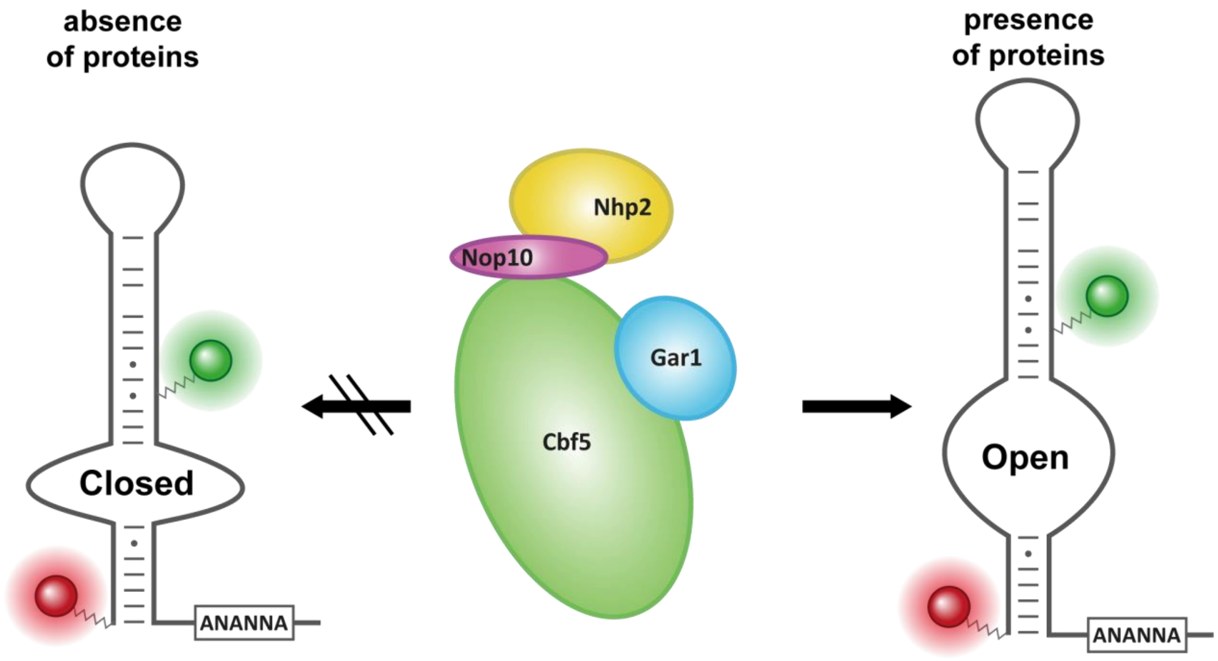

Figure S9: Schematic representation of pseudouridylation pocket conformation in absence (closed) or presence (open) of the Nhp2-Nop10-Cbf5-Gar1 protein complex. We assume the low-FRET state to resemble the "open" and the intermediate-FRET state the "closed" conformation of H5.

Supplementary Figure S10

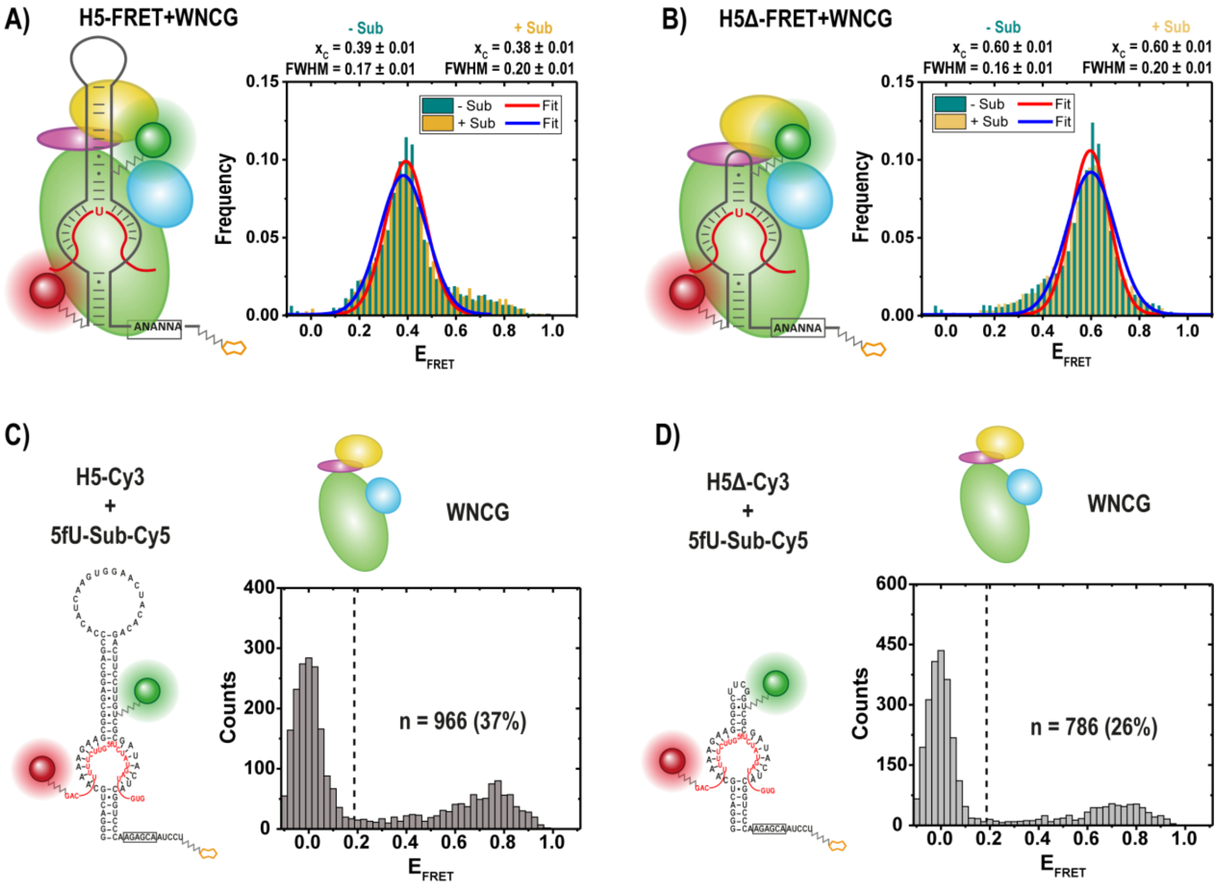

Figure S10: Substrate addition to fully assembled complexes. A) Histograms of H5-FRET+WNCG in absence (green) and presence (yellow) of substrate RNA. B) Histograms of H5Δ -FRET+WNCG in absence (green) and presence (yellow) of substrate RNA. Substrate RNA was present in excess (2 µM) during imaging. Percentages are derived from fitting of Gaussian distributions. No significant shifts in E_{FRET} upon substrate addition were observed.

Substrate binding tests for Cy3 labeled H5 C) and Cy3 labeled H5Δ D) in presence of WNCG were preannealed with 1.3 molar eq. of Cy5 labeled substrate analogue (5fU-Sub-Cy5) carrying 5-fluoro-uracil (5fU) as target nucleotide. No additional 5fU containing substrate analogue RNA was present during imaging. n represents the number of molecules observed for $E_{FRET} \geq 0.2$. For both constructs distinct substrate binding was observed.

For detecting whether bonafide substrate RNA induces a conformational shift, we added an approximately 10000-fold excess of substrate RNA. This did not lead to a detectable shift in E_{FRET}, indicating that binding of substrate RNA does not induce a conformational change. To verify that our RNPs are indeed substrate binding competent we used a 5fU containing substrate analogue with identical base pairing sequences in near equimolar quantities to the RNP. 5fU was used for these experiments, since it inhibits pseudouridine synthases (3–6) and likely results in a more stable target RNA bound complex.

Supplementary Figure S11

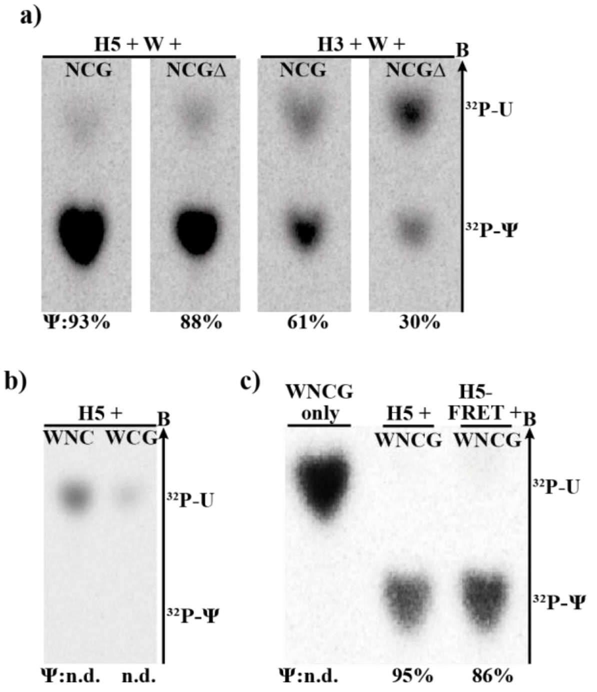

Figure S11: Thin layer chromatography analysis of single turnover pseudouridylation activity tests of standalone hairpins, and negative control experiments. a) H5 and H3 with full RNP and with Gar1Δ. Both hairpins show high enzymatic activity and reduced activity in the absence of RGG domains. b) H5 RNP in the absence of Gar1 or Nop10. Both proteins are necessary for catalytic activity. c) Activity in absence of guide RNA ("WNCG only") as well as FRET-labeled H5 in comparison to non-labeled H5. Proteins do not exhibit activity on their own, and labeling does not inhibit catalytic activity.

Supplementary Figure S12

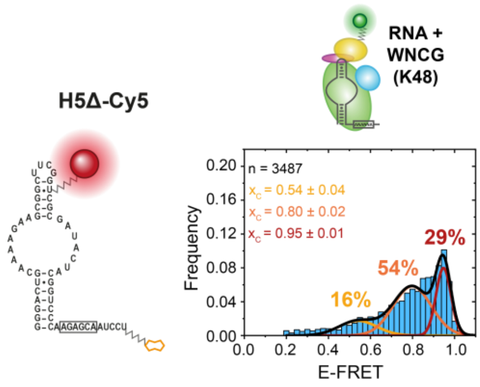

Figure S12: Binding experiment of donor-labeled Nhp2 and acceptor labeled H5Δ in the fully assembled RNP. n represents the number of molecules observed for E-FRET ≥ 0.2. Percentages are derived from fitting of Gaussian distributions.

A rather broad FRET population is visible, clearly showing that Nhp2 still interacts with the RNP even in absence of the apical binding motif. Upon further analysis of this experiment with time-resolved FRET traces as well as HaMMy analysis, we could actually pinpoint three distinct FRET populations for this experiment (E_{FRET}=0.54, 0.80 and 0.95). This demonstrates that in absence of the apical RNA binding motif in H5Δ, the association of Nhp2 to the RNP is not as straightforward as for H5, where only a single binding mode could be observed (Figure 4a). Without the RNA binding motif, there rather seems to be three distinct binding modes of Nhp2, similar to the FRET results of construct H3 (Figure 4b). However, this is even more remarkable, since the disruption of the single binding mode of Nhp2 is accompanied by an increase in activity (Figure 3c).

These observations can lead to two different conclusions: Either all observable binding modes of Nhp2 in this experiment contribute to an activity increase of the complex and represent an active RNP, or only one of the observed binding modes leads to an activity increase, while the other two observable binding modes represent inactive or (partly) misfolded RNPs. However, in the second case, even in the presence of the inactive/misfolded conformations, there would still be an activity increase in the presence of Nhp2.

Since only RNP molecules containing fluorophore labeled Nhp2 are detected in this experiment (and no complexes with only NCG), we however cannot directly compare the affinity of Nhp2 to H5Δ vs. H5.

Supplementary Figure S13

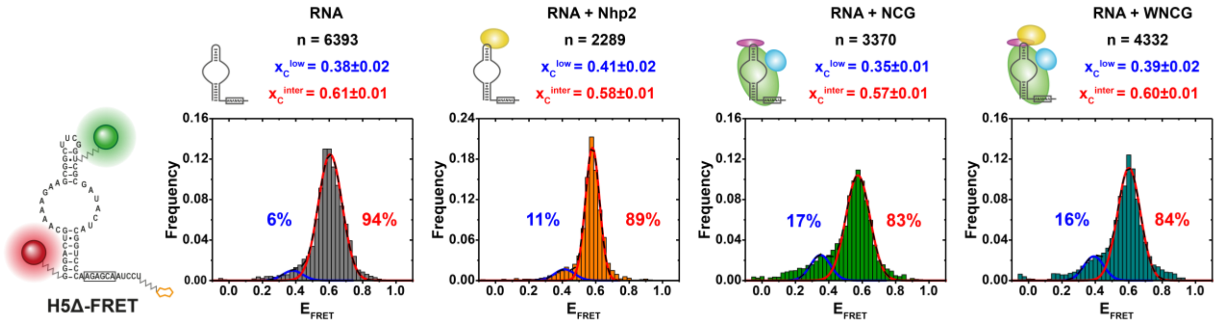

Figure S13: Analysis of H5Δ assembly, comparing histograms for (from left to right): RNA only, RNA with Nhp2, RNA with Nop10, Cbf5, and Gar1 ("NCG"), and RNA with NCG and Nhp2 ("WNCG"). Percentages are derived from fitting of Gaussian distributions. Construct H5Δ showed no major transition from the intermediate (centered at x_c^{inter}≈0.60) to the low FRET state (centered at x_c^{low}≈0.38) upon addition of proteins.

Supplementary Figure S14

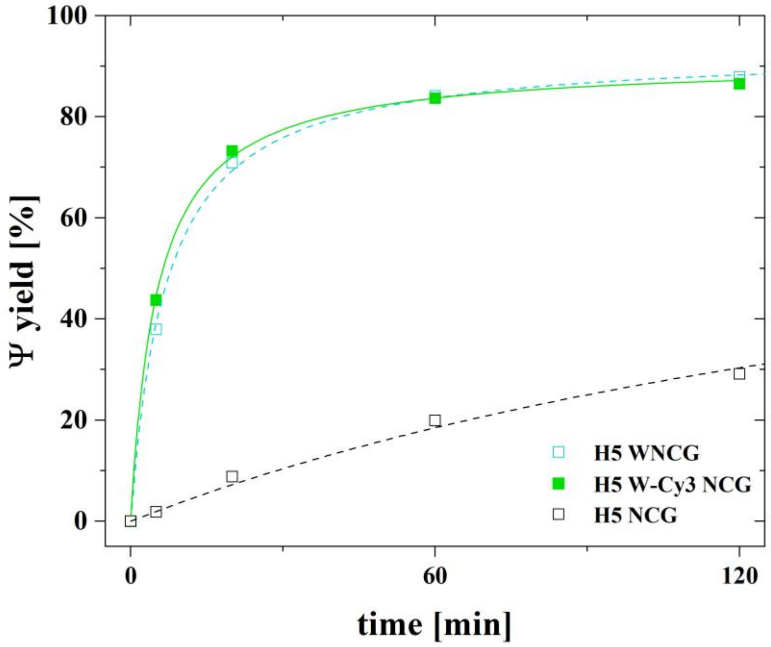

Figure S14: Time course of pseudouridylation reaction under multiple turnover conditions of H5 snoRNA with Nhp2K48Cy3 (W-Cy3) and NCG (green) in comparison to WNCG (cyan) and NCG (black). Labeling of Nhp2 does not inhibit catalytic activity.

Supplementary Figure S15

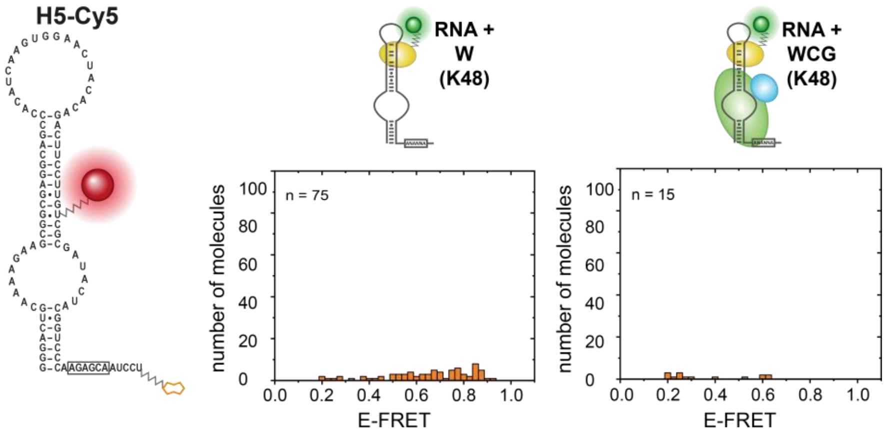

Figure S15: a) Histogram of 5’ hairpin complex H5-Cy5 + Nhp2 K48Cy3 (omission of NCG). b) Histogram of 5’ hairpin complex H5-Cy5 + Nhp2 K48Cy3 + CG (omission of Nop10). n represents the number of molecules observed for E-FRET ≥ 0.2. No populations can be observed for the two control experiments.

Supplementary Figure S16

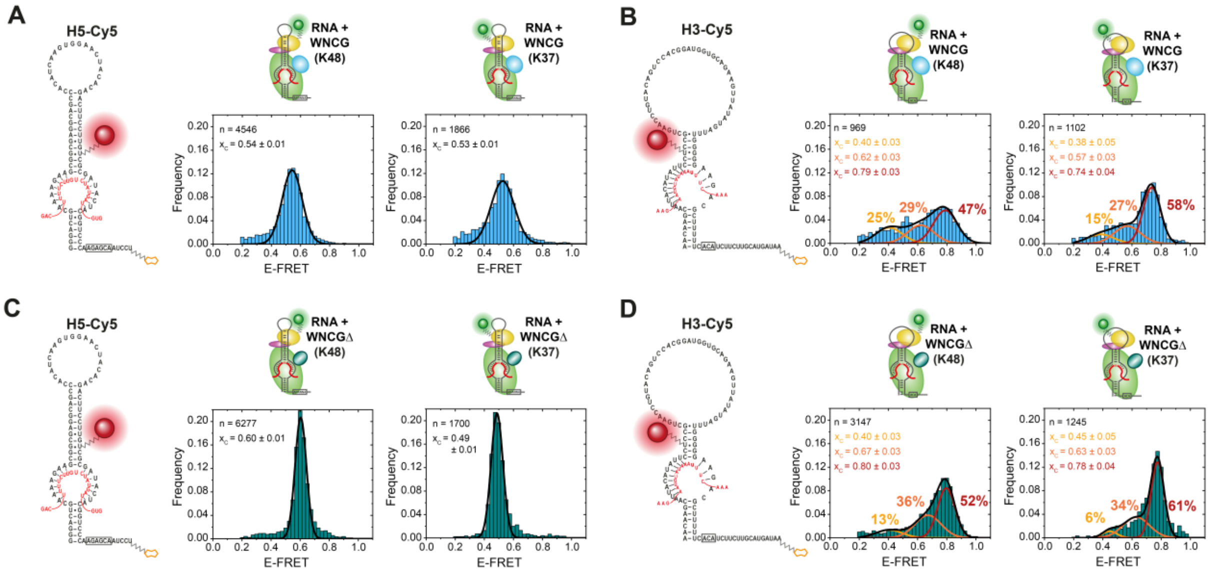

Figure S16: Substrate addition to fully assembled complexes. A) Histograms of H5-Cy5 + NCG + Nhp2K48-Cy3 (left) and Nhp2K37-Cy3 (right). B) Histograms of H3-Cy5 + NCG + Nhp2K48-Cy3 (left) and Nhp2K37-Cy3 (right). C) Histograms of H5-Cy5 + NCGΔ + Nhp2K48-Cy3 (left) and Nhp2K37-Cy3 (right). D) Histograms of H3-Cy5 + NCGΔ + Nhp2K48-Cy3 (left) and Nhp2K37-Cy3 (right). Substrate RNA was present in excess (100 nM) during imaging. n represents the number of molecules observed for E-FRET ≥ 0.2. Percentages are derived from fitting of Gaussian distributions. No significant shifts in E_{FRET} upon substrate addition were observed for all experiments, except for H3-Cy5 + NCG + Nhp2K37-Cy3 (B, right side), where a shift of populations from the middle E_{FRET} state towards the high E_{FRET} state of ~ 15% could be observed (compared to Figure 4C, right side).

References

1. Schmidt, A., Altincekic, N., Gustmann, H., Wachtveitl, J. and Hengesbach, M. (2018) The Protein Microenvironment Governs the Suitability of Labeling Sites for Single-Molecule Spectroscopy of RNP Complexes, *ACS chemical biology,* **13,** 2472–2483.
2. Kalinin, S., Peulen, T., Sindbert, S., Rothwell, P.J., Berger, S., Restle, T., Goody, R.S., Gohlke, H. and Seidel, C.A.M. (2012) A toolkit and benchmark study for FRET-restrained high-precision structural modeling, *Nature methods,* **9,** 1218–1225.
3. Samuelsson, T. (1991) Interactions of transfer RNA pseudouridine synthases with RNAs substituted with fluorouracil, *Nucleic acids research,* **19,** 6139–6144.
4. Patton, J.R. (1993) Ribonucleoprotein particle assembly and modification of U2 small nuclear RNA containing 5-fluorouridine, *Biochemistry,* **32,** 8939–8944.
5. Huang, L., Pookanjanatavip, M., Gu, X. and Santi, D.V. (1998) A conserved aspartate of tRNA pseudouridine synthase is essential for activity and a probable nucleophilic catalyst, *Biochemistry,* **37,** 344–351.
6. Zhao, X. and Yu, Y.-T. (2007) Incorporation of 5-fluorouracil into U2 snRNA blocks pseudouridylation and pre-mRNA splicing in vivo, *Nucleic acids research,* **35,** 550–558.
